# Species-specific chlorine resistance and biofilm regulation by extracellular polymeric substances and quorum sensing in drinking water pipeline bacteria

**DOI:** 10.1128/aem.01531-25

**Published:** 2026-03-13

**Authors:** Jia Niu, Daogan Chen, Tian Lin, Shixuan Li, Jiayan Xu, Huacheng Xu, Xianhua Liu, Xiaochen Chen, Nan Wei, Kro Meng

**Affiliations:** 1 Fujian Engineering Research Center of Water Pollution Control and System Intelligence Technology, School of Ecological Environment and Urban Construction, Fujian University of Technology 34738, Fuzhou, Fujian, People's Republic of China; 2 College of Materials Science and Engineering, Huaqiao University, Xiamen, Fujian, People's Republic of China; 3 Quanzhou Lanshen Environmental Protection Research Institute Co. Ltd., Quanzhou, People's Republic of China; 4 Fujian Lanshen Environmental Protection Technology Co. Ltd., Quanzhou, People's Republic of China; 5 School of Environmental Science and Engineering, Tianjin University 12605, Tianjin, People's Republic of China; 6 College of Environment and Safety Engineering, Fuzhou University 12423, Fuzhou, People's Republic of China; 7 State Key Laboratory of Soil Pollution Control and Safety, Chinese Academy of Environmental Planning 90404, Beijing, People's Republic of China; 8 Department of Environmental Science, Royal University of Phnom Penh 106092, Phnom Penh, Cambodia; Indiana University Bloomington, Bloomington, Indiana, USA

**Keywords:** drinking water distribution system, biofilm, extracellular polymeric substances, chlorine resistance, quorum sensing

## Abstract

Biofilms in drinking water distribution systems pose significant risks by harboring chlorine-resistant bacteria. This study isolated five bacterial strains (*Sphingomonas ursincola*, *Sphingobium amiense*, *Gordonia amicalis*, *Microbacterium saccharophilum*, *Hydrogenophaga laconesensis*) from municipal pipelines to evaluate differences in biofilm formation and chlorine resistance. Biofilm formation ability varied notably, with *S. ursincola* showing the strongest capacity and *H. laconesensis* the weakest. Quorum sensing (QS) signal molecules (C_6_-HSL, 3-OXO-C_14_-HSL; 0.2240–0.2481 μg/L) and extracellular polymeric substances (EPSs: 19.940–32.407 mg/L) were critical in biofilm regulation, where QS molecules influenced EPS composition. Chlorine resistance assays revealed species-specific tolerance: at ≤1.0 mg/L, resistance ranked *M. saccharophilum* > *S. amiense* > *G. amicalis* > *S. ursincola* > *H. laconesensis*; above 1.0 mg/L, *S. ursincola* and *S. amiense* exhibited robust resistance. Low chlorine levels (0.6 mg/L) damaged only 25.91%–34.80% of bacteria, insufficient to control the biofilm formation of the tested isolates. Optimal disinfection occurred at 1.0–1.5 mg/L, effectively controlling biofilm biomass. These findings highlight EPS-driven chlorine resistance mechanisms and QS-mediated biofilm regulation, providing actionable strategies for pipeline management.

**IMPORTANCE:**

This study addressed a critical gap in understanding different bacterial biofilm dynamics and chlorine resistance mechanisms in drinking water systems. By linking quorum sensing to extracellular polymeric substance production, it reveals how bacteria modulate biofilm resilience, elucidating species-specific mechanisms underlying biofilm resilience to chlorine disinfection. The identification of chlorine-resistant species (*Sphingomonas ursincola*, *Sphingobium amiense*) and optimal disinfection thresholds (1.0–1.5 mg/L) directly informs municipal water treatment protocols, providing a practical chlorine concentration range (1.0–1.5 mg/L) that effectively controls biofilms while avoiding excessive disinfectant use. These results are pivotal for mitigating secondary contamination risks and safeguarding public health, particularly in aging infrastructure where biofilm-related outbreaks are prevalent.

## INTRODUCTION

Biostability of drinking water is crucial for ensuring public health. In recent years, to meet the growing demand for high-quality drinking water, China updated the standards for drinking water quality (GB5749-2022), strengthening the requirements of microbial control. Drinking water distribution systems (DWDSs) play a key role in the transportation and delivery of high-quality drinking water (1). In DWDSs, microorganisms exist in the bulk water (only 5% of the total amount), and biofilms form on the inner surfaces of pipes (95% of the total amount) (2). These pipeline biofilms can consume disinfectants, reduce the disinfection effects, and become a refuge for pathogens (3). Biofilms provide a relatively stable environment for microorganisms to grow even under the oligotrophic conditions typical of DWDSs (4), resulting in water quality-related issues such as discoloration, turbidity, and odor, as well as in pipeline corrosion (5). Therefore, it is crucial to understand the mechanisms through which bacteria form biofilms within pipelines to develop effective biofilm-control strategies.

Although next-generation sequencing techniques (e.g., high-throughput sequencing and metagenomic analysis [6, 7]) can be used to detect the presence of biofilm microorganisms in DWDSs as well as their diversity, they cannot distinguish between viable and non-viable cells (8, 9). As a result, when examining biofilm formation and growth characteristics, the functional roles of detected microorganisms may be misinterpreted, potentially leading to an over- or underestimation of their contributions to biofilm development. Consequently, detailed investigations into the biofilm formation process and the growth characteristics of individual bacterial strains remain limited. Moreover, most existing studies have focused primarily on biofilm development, without addressing chlorine resistance or the role of signaling molecules in biofilm formation (10–13). Therefore, a comprehensive understanding of biofilm formation in DWDSs and its implications for microbial ecology is still lacking.

As bacteria adhere to the pipeline walls and grow, they secrete extracellular polymeric substances (EPSs) to enhance cell adhesion (14, 15), thus strengthening the connection among bacteria within the biofilm and temporarily fixing their position. This secretion improves the biofilm’s resistance to the shear force of the water flow (16), which explains why biofilms are not easily removed from pipe walls in DWDSs by scouring (17, 18). Microorganisms can regulate EPS production through signal molecules, that is, *N*-acyl-homoserine lactones (AHLs), during quorum sensing (QS) to enhance biofilm formation (19). However, most previous studies have focused on the effect of one type of signal molecule secreted by a single strain on the EPS of microbial communities, not considering the effect of signal molecules secreted by various strains on the whole microbial community (20–22).

In the real-world setting, the main way of controlling microbial regeneration in DWDSs is by disinfecting the finished water via the addition of chlorine (12). However, some studies have reported that chlorine application was not sufficiently effective in inhibiting some bacteria, especially the chlorine-resistant ones (13, 23). Although chlorine disinfection suppresses microbial proliferation in distribution networks, the potential for subsequent regrowth remains a persistent challenge (24). Moreover, microbial reactivation occurs among antibiotic-resistant pathogens after exposure to low-concentration chlorine residuals, thus creating substantial public health risks (25). Therefore, the appropriate range of chlorine concentrations should be scientifically established based on the resistance of different microorganisms to this disinfectant. Furthermore, due to the new limits for the maximum concentration of free chlorine (2.0 mg/L) in finished water set by China’s national standard for drinking water quality (GB5749-2022), it is necessary to reevaluate the chlorine concentrations for the effective control of biofilm regrowth.

In this study, bacteria were isolated from biofilm samples collected from the inner wall of two kinds of pipelines within DWDSs. Biofilm formation of each bacterium was analyzed in 96-well microtiter plates. The biofilm formation ability (BFA) of different isolated bacteria was analyzed and evaluated. Furthermore, the biofilms produced by the five strains were subjected to a chlorine disinfection experiment to investigate their mechanism of resistance. The results of this study contribute to elucidating the biofilm formation process from the perspective of single-species biofilms, providing valuable references for controlling biofilm regrowth via chlorine addition.

## MATERIALS AND METHODS

### Biofilm sampling and pretreatment

Biofilm sampling and pretreatment were conducted based on the methods described in Chen et al. (26). Pipe scales and biofilms were collected from two kinds of pipe sections (gray cast iron pipe and ductile iron pipe) that have been used for more than 10 years. Information regarding the pipelines and the water quality of the inlet and outlet is presented in Table S1. The pipe sediment and scales on the pipe wall were scraped with a sterilized stainless-steel spoon, collected into a 50 mL sterile centrifuge tube, and added to 30 mL of sterile phosphate-buffered saline (PBS, pH 7.2). The sample was then placed on ice and quickly transferred to the laboratory, where it was stored in a refrigerator at 4°C.

The collected biofilm sample was transferred to an aseptic conical bottle containing a sufficient amount of sterile glass beads. Subsequently, the sealed conical bottle was shaken for 20 min at a speed of 100 rpm in a 25°C incubator. Next, the solution was filtered through a sterilized 80-mesh screen to remove impurities and rinsed with sterile PBS (pH 7.2) three times. The filtered slurry was transferred to an aseptic centrifuge tube and centrifuged at 4,500 rpm for 15 min. Finally, after removing the supernatant, the precipitate was resuspended in sterile PBS (pH 7.2).

### Isolation and identification of the biofilm bacteria

The pretreated bacterial suspensions were incubated using the R2A, LB, and 337 media (12, 27, 28). The composition of Medium 337 is as follows (27): 1.3 g KH_2_PO_4_, 2.13 g Na_2_HPO_4_, 0.5 g (NH_4_)_2_SO_4_, 0.2 g MgSO_4_·7H_2_O, 5.97 mg CaCl_2_·2H_2_O, 3.0 mg FeSO_4_·7H_2_O, 1.5 mg Na_2_MoO_4_·2H_2_O, and 1.05 mg MnSO_4_·4H_2_O, dissolved in 1,000 mL of distilled water. Methanol (0.5% vol/vol) and vitamin B_12_ (0.1 mg/L) were then added to the medium. The medium was solidified with 1.8% (wt/vol) Bacto agar (28). The bacteria present in the biofilms were isolated using the gradient dilution method. To isolate as a variety of bacteria as possible, the incubation conditions were adjusted to pH 7.0, 7.2, and 7.4, and the corresponding temperatures for each pH were 25°C, 30°C, and 37°C, respectively. All the plates were incubated in dark conditions for 3–7 days.

The extraction of DNA from the isolated bacteria was performed using Ezup Column Bacteria Genomic DNA Purification Kit (SK8255, Sangon Biotech Co., Ltd., China) in accordance with the manufacturer’s protocol. Then, the extracted DNA was sent to Sangon Biotech (Shanghai, China) for sequencing of the full length of the 16S rRNA gene of the isolated bacteria.

### Bacterial growth curve

The isolated strains were individually inoculated into R2A liquid medium, with the pH adjusted to 7.0. The cultures were incubated at 25°C under continuous shaking at 150 rpm in the dark (29). The absorbance (OD_600_) of the bacterial cultures was measured at 600 nm using an ultraviolet spectrophotometer (T6, Beijing Purkinje General Instrument Co., Ltd., China). The obtained values were then fitted to an *SLogistic1* function to construct growth curves, which allowed for the determination of microbial growth phases, including the adaptation period, logarithmic phase, stationary phase, and decline phase (29–31).

### Biomass, activity, and BFA of the isolated biofilm bacteria

The biofilms were incubated in a 96-well microtiter plate, based on the methods described in Stepanovic et al. (32) and Simoes et al. (33), with some modifications. This simplified biofilm model is a useful tool to understand the effect of a specific bacterium on the entire community and to compare the biofilm formation process, biofilm formation ability, and chlorine tolerance of different bacteria (10). Additionally, this method allows associating a certain molecule with a certain bacterial strain (11). A total of 180 μL of diluted bacterial solution was added to the sterile 96-well microtiter plates (Corning Incorporated, USA). Only 180 μL of R2A medium without bacteria was used as the negative control. The 96-well microtiter plates were incubated at 25°C under shaking at 150 rpm for 24, 48, and 72 h. Every 24 h, the fresh R2A liquid medium was replaced using a pipette in a clean bench during the incubation period. The medium was removed, and the plates were then rinsed gently with 250 μL of sterile PBS (pH 7.0) thrice to remove unattached or loosely attached bacteria. The culture plates were air-dried for 30 min before conducting further analyses.

The biomass of the obtained biofilms was determined based on the method described by Stepanovic et al. (32), with some modifications. After air-drying, the biofilm sample in each well was fixed with 180 μL of 98% (vol/vol) methanol for 15 min. Following fixing, the methanol was discarded. Then, 180 μL of 1% crystal violet (CV) dye was added to each well for 5 min. The dyed biofilm was cleaned with aseptic distilled water to remove the excess dye and then air-dried. Subsequently, 180 μL of 33% (vol/vol) glacial acetic acid was added to each well to release and dissolve the CV. The absorbance value of each well was measured using an enzyme-labeling instrument (SPECTROstar Nano, BMG LABTECH, Germany) at a wavelength of 570 nm (OD_570_). The experiment was conducted in triplicate.

Biofilm activity was determined according to the method described by Simões et al. (34). Briefly, 200 μL of the sodium 3,3’-[1[(phenylamino)carbonyl]-3,4-tetrazolium]-bis(4-methoxy-6-nitro)benzene sulfonic acid hydrate (XTT, Sigma, USA) and menadione (Sigma, USA) solutions were added into each biofilm to obtain a final concentration of 50 μg/mL XTT. The plates were incubated on a shaker at 150 rpm at 25°C for 3 h in the dark. Then, 90 μL of supernatant from each plate was transferred into a new microtiter plate. The absorbance of each well was determined at a wavelength of 490 nm (OD_490_) using the same enzyme-labeling instrument. Biofilm activity was determined at OD_490/570_ to specifically reflect bacterial respiration (35). The bacteria’s BFA was determined based on the method described in Stepanovic et al. (32). The standard deviation three times greater than the average OD_570_ value of the negative control (3SD) was defined as the critical value (cutoff value, OD_C_). Based on this value, four BFA levels were identified: no biofilm formation potential (0): OD_570_ ≤ OD_C_; weak biofilm formation potential (+): OD_C_ < OD_570_ ≤ 2 OD_C_; medium biofilm formation potential (++): 2 OD_C_ < OD_570_ ≤ 4 OD_C_; and strong biofilm formation potential (+++): OD_570_ > 4 OD_C_.

### EPS content of the biofilms

The biofilm-produced EPSs were extracted using the heating method (36, 37). In brief, the biofilm samples formed by the five bacterial strains at different incubation periods (24 h, 48 h, and 72 h) were separately transferred into 2 mL sterile microcentrifuge tubes containing 1 mL of sterilized 0.9% NaCl solution. The microcentrifuge tubes were centrifuged at 4,000 r/min for 5 min at 4°C. The supernatant was collected after filtration with a 0.45 μm cellulose acetate membrane, and the biofilm was resuspended in the same volume of aseptic 0.9% NaCl solution and centrifuged at 8,000 r/min for 10 min at 4°C. The supernatant was collected after repeating the previous steps. Finally, after repeated resuspension in the same volume of aseptic 0.9% NaCl solution, the supernatant was heated in a water bath at 70°C for 30 min. Centrifugation was then repeated at 8,000 r/min, and the supernatant was collected in the same way. The supernatant obtained through successive rounds of centrifugation was mixed with the prior collections for subsequent quantification of EPS constituents. The Bradford method (with some modifications) and the anthrone-sulfuric acid method were used to determine the protein and polysaccharide contents in the EPSs (38, 39), respectively (via a T6 ultraviolet spectrophotometer, Beijing Purkinje General Instrument Co., Ltd., China).

### Chlorine resistance experiment

NaClO diluted with PBS (pH 7.0) was used as the chlorine solution (40, 41), as this is a common disinfectant used in drinking water treatment. The concentration of free chlorine in drinking water was adjusted according to the new standards for drinking water quality (GB5749-2022), which establish that the maximum free chlorine concentration in finished and tap water cannot exceed 2 mg/L. The dilution with PBS was carried out to obtain chlorine concentrations of 0.3, 0.6, 1.0, 1.5, and 2.0 mg/L.

Biofilm samples formed by five individual bacterial species, following 24 h and 72 h of incubation, were subjected to the chlorine resistance test (42). After being washed with sterile PBS (pH 7.0), each sample was added with 180 μL of chlorine solution at one of the above-mentioned concentrations. In this test, 180 μL of sterile PBS and a biofilm sample cultured with 180 μL of chlorine-free sterile PBS were used as negative and positive controls, respectively. The test was carried out at 25°C under shaking at 150 rpm. According to the new standards for drinking water quality (GB5749-2022), when using NaClO as the disinfectant, the contact time in water should be ≥30 min; therefore, the contact time was set to 1 h. During the disinfection, fresh chlorine disinfectant was gently introduced every 20 min (12). After disinfection, the plates were rinsed twice with 180 μL of 0.5% (wt/vol) Na_2_S_2_O_3_ solution to remove excess chlorine. Next, 180 μL of sterile PBS was added to rinse the excess Na_2_S_2_O_3_ solution and remove any loosely attached bacteria in each plate. The plates were then air-dried for 30 min before conducting further analyses.

The disinfection efficiency of chlorine was calculated using two parameters: the reduction in biofilm biomass and the logarithmic reduction of biofilm bacteria, as shown in equations 1 and 2 (12, 43):


(1) $$\text{Reduction}\;\text{in}\;\text{biofilm biomass}\text{=}\left(\text{1-}\frac{{\text{OD}}_{\text{570}}\text{(chlorination)-}{\text{OD}}_{\text{570}}\text{(negative control)}}{{\text{OD}}_{\text{570}}\text{(positive control)-}{\text{OD}}_{\text{570}}\text{(negative control)}}\right)\times 100\%$$

(2) $$\text{L}\text{ogarithm}\text{ic}\;reduction\;of\;biofilm\;bacteria\;=\;log_{\text{10}}\left(\frac{{\text{N}}_{\text{0}}}{\text{N}}\right)\text{=}\;log_{\text{10}}\left(\frac{\text{bacterial counts (positive control)}}{\text{bacterial counts (chlorination)}}\right)$$

An absorption value measured by an enzyme-labeling instrument (SPECTROstar Nano, BMG LABTECH, Germany) at a wavelength of 570 nm (OD_570_) for the chlorinated experimental group that was lower than that for the negative control group indicated a 100% reduction in biofilm biomass.

After incubation, the biofilm samples were washed and dispersed with 1.8 mL of aseptic distilled water, transferred to 2 mL aseptic microcentrifuge tubes, and then placed into sterile flow tubes. Finally, the number of bacteria was determined by flow cytometry (NovoCyte Advanteon BR, Agilent Technologies, Inc., USA) equipped with a 488 nm solid-state laser. Following the method described by Berney et al. (44), cells were subjected to live/dead staining using propidium iodide (PI; Sigma, USA) and SYBR Green I (Invitrogen, Switzerland), with appropriate optical filters set on the flow cytometer. SYBR Green I is a membrane-permeant dye that stains all cells by binding to DNA, emitting green fluorescence at 520 nm when excited at 488 nm. Conversely, PI is a membrane-impermeant dye that can only enter cells with compromised membrane integrity. Upon entry, it binds to nucleic acids, yielding red fluorescence at 635 nm. This differential staining enables the discrimination of distinct cell populations based on their membrane status (1, 45). For each sample, 500 μL was incubated with 5 μL of SYBR Green I (100× diluted in anhydrous dimethyl sulfoxide) and PI at a final concentration of 4 µg/mL. The stained samples were then incubated in the dark at 30°C for 15 min prior to flow cytometric analysis. All dye stocks were stored at −20°C.

### Extraction and detection of AHLs

Signal molecules, that is, AHLs, were extracted based on the methods described by Tang et al. (46) and McClean et al. (47). The five isolated bacterial strains were individually inoculated into R2A liquid medium, with the pH adjusted to 7.0. The cultures were then incubated under continuous shaking at 150 rpm and 25°C in the dark for 3–5 days. After being incubated up to the steady stage, 20 mL of bacterial culture was centrifuged at 4,500 rpm for 15 min. The supernatant was removed and placed into a sterilized conical bottle and then mixed with the same volume of ethyl acetate (Sigma, USA). After sealing, ultrasonic concussion was carried out for 10 min; then, the sealed bottle was shaken at 300 rpm for 10 min. After this step, the ethyl acetate phase was collected using a funnel for liquid separation, and the water phase was added with the same volume of ethyl acetate and oscillated. After repeating the extraction process three times with ethyl acetate, the sample was dried using nitrogen gas. The remaining white substance in the sample bottle was dissolved in 2 mL of a methanol-water mixture (methanol high-performance liquid chromatography grade, ultrapure water, 0.1% formic acid). The sample was filtered through a 0.22 μm organic microporous membrane, placed into a 2 mL chromatographic injection bottle, and stored at −20°C for subsequent detection of signal molecules.

In total, 11 types of common AHL standards (Shanghai Macklin Biochemical Technology Co., Ltd., China) (48) were dissolved in a pure methanol solution for chromatographic analysis. The AHLs were analyzed by high-performance liquid chromatography-mass spectrometry (48) using an advanced benchtop tandem quadrupole mass spectrometer (Agilent 1290 Infinity II 6470B, Agilent Technologies, Inc., USA) and a C18 column (Eclipse Plus, Agilent Technologies, Inc., USA). The mobile phase A was ammonium acetate (4 mmol/L ammonium acetate, 0.1% formic acid), phase B was methanol, and a gradient elution with a flow rate of 0.3 mL/min was used during the separation process, as shown in Table S2. The optimized mass spectrometry parameters in multiple reaction monitoring mode are shown in Table S3.

### Impact of AHL supplementation on biofilm formation and EPS production

To investigate the impact of AHLs on the formation of biofilms by the five bacterial strains, three exogenous AHLs—C_6_-HSL, C_14_-HSL, and 3-OXO-C_14_-HSL—were supplemented during biofilm cultivation. Briefly, during the biofilm formation process, each AHL was supplemented at varying concentrations (0.05 μg/L, 0.10 μg/L, 0.50 μg/L, and 1.00 μg/L). A blank control group was maintained with sterile PBS. The cultivation conditions were consistent with those used for prior biofilm growth, including R2A liquid medium, pH adjusted to 7.0, and incubation under continuous shaking at 150 rpm and 25°C in the dark for 24 h. The biofilms obtained from this cultivation were subsequently used for EPS quantification.

### Statistical analysis

The data were statistically analyzed using one-way analysis of variance and Pearson correlation analysis (35) (SPSS 26 Statistic, IBM Corp., USA). Statistical calculations were based on a confidence level ≥95%, assuming a significance level for the separation set at *P* < 0.05.

## RESULTS

### Characteristics of the bacteria isolated from the DWDS

**Fig 1:**
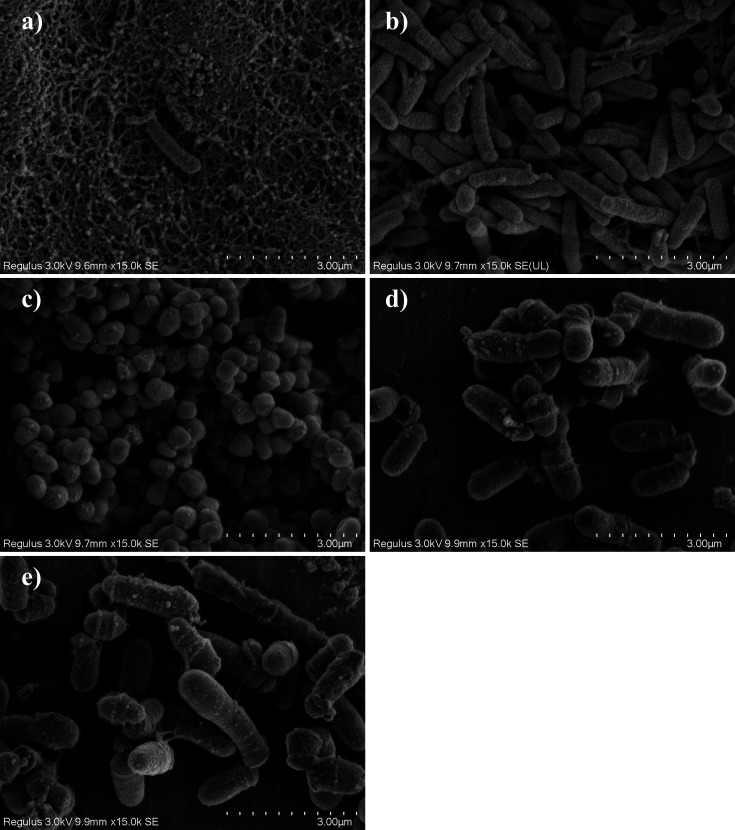
Scanning electron microscope images of the strains (15,000×). (**a**) *Hydrogenophaga laconesensis* (gram-negative, long rod-shaped, approximately 1.2 µm in length and 0.3 µm in diameter). (**b**) *Sphingobium amiense *(a gram-negative, rod-shaped, approximately 1.5 µm in length and 0.3 µm in diameter). (**c**) *Sphingomonas ursincola* (gram-negative, short rod-shaped or coccoid morphology, 0.6 to 0.9 µm in length and approximately 0.3 µm in diameter). (**d**) *Microbacterium saccharophilum* (a rod-shaped, gram-positive, 0.9 to 1.2 µm in length and approximately 0.6 µm in diameter). (**e**) *Gordonia amicalis* (gram-positive, rod-shaped, 0.6 µm–1.5 µm in length and approximately 0.6 µm in diameter).

Five bacterial strains were isolated from the pipeline biofilm sample and identified as *Hydrogenophaga laconesensis* (*Hyd*), *Sphingobium amiense* (*Spb*), *Sphingomonas ursincola* (*Spm*), *Gordonia amicalis* (*Gor*), and *Microbacterium saccharophilum* (*Mic*). Images of the strains obtained via scanning electron microscopy are illustrated in Fig. 1, and more detailed information on them is provided in Supplementary Information Table S4.

**Fig 2:**
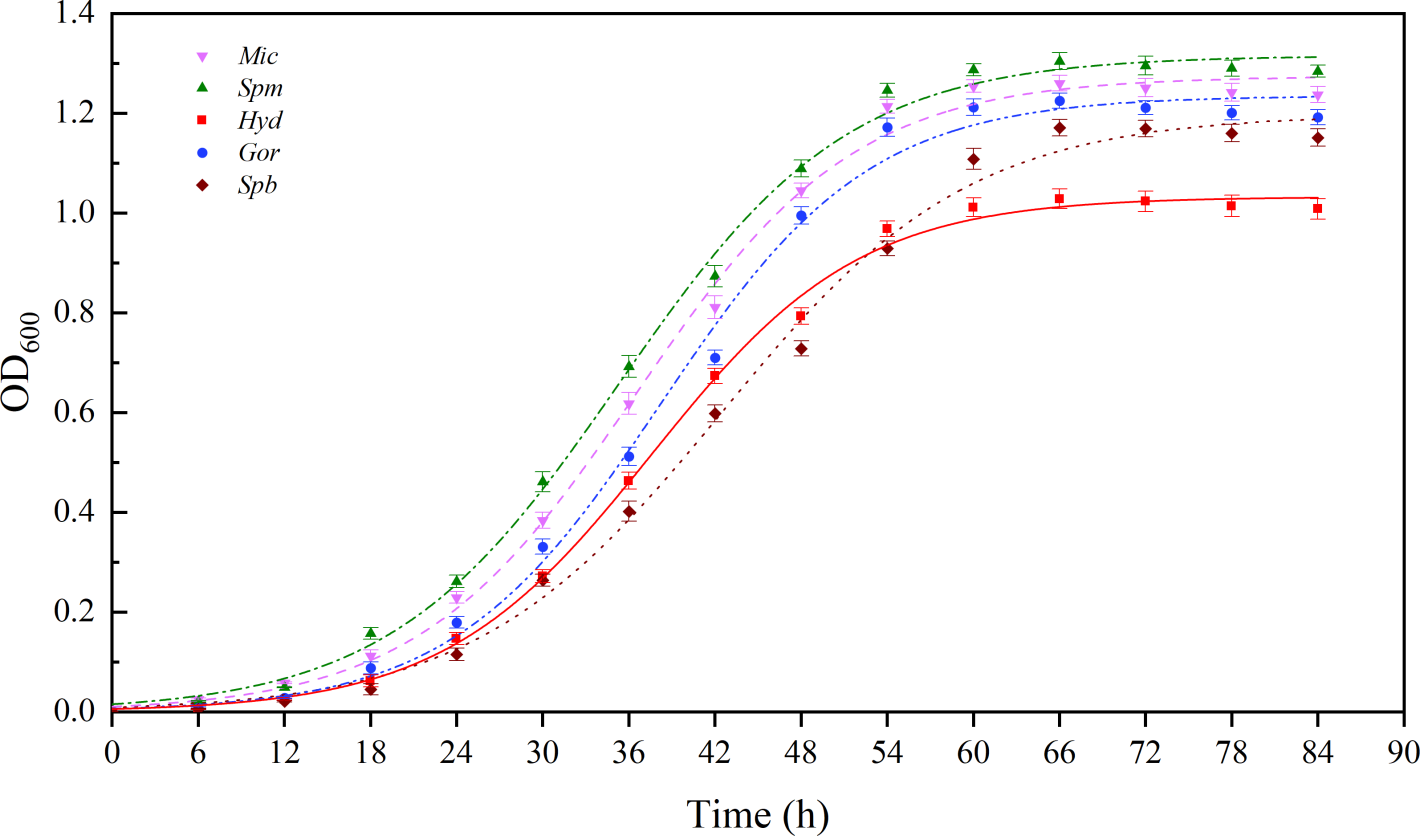
Growth curves for the bacteria isolated from the DWDS. The data are expressed as the mean ± SEM. *Gor*, *Gordonia amicalis*; *Spb*, *Sphingobium amiense*; *Hyd*, *Hydrogenophaga laconesensis*; *Spm*, *Sphingomonas ursincola*; *Mic*, *Microbacterium saccharophilum*.

Morphologically, the five strains had the shapes of cocci, short rods, and long rods, as shown in (Fig. 1). The cocci and short rods were relatively small, with diameters ranging from 0.3 to 0.6 μm and lengths of ≤1 µm, respectively, while some long rods, for example, in the cases of *Hyd* and *Gor*, exceeded 1 μm in length. The bacterial growth curve (Fig. 2) showed that the five strains were in the adjustment period during the first 12 h of incubation, exhibiting a low growth rate. However, the logarithmic growth phases of *Spm*, *Mic*, *Hyd,* and *Gor* lasted from 12 to 54 h, and that of *Spb* was even longer (from 12 to 66 h).

### Changes in the biofilm biomass of the five isolated bacteria

**Fig 3:**
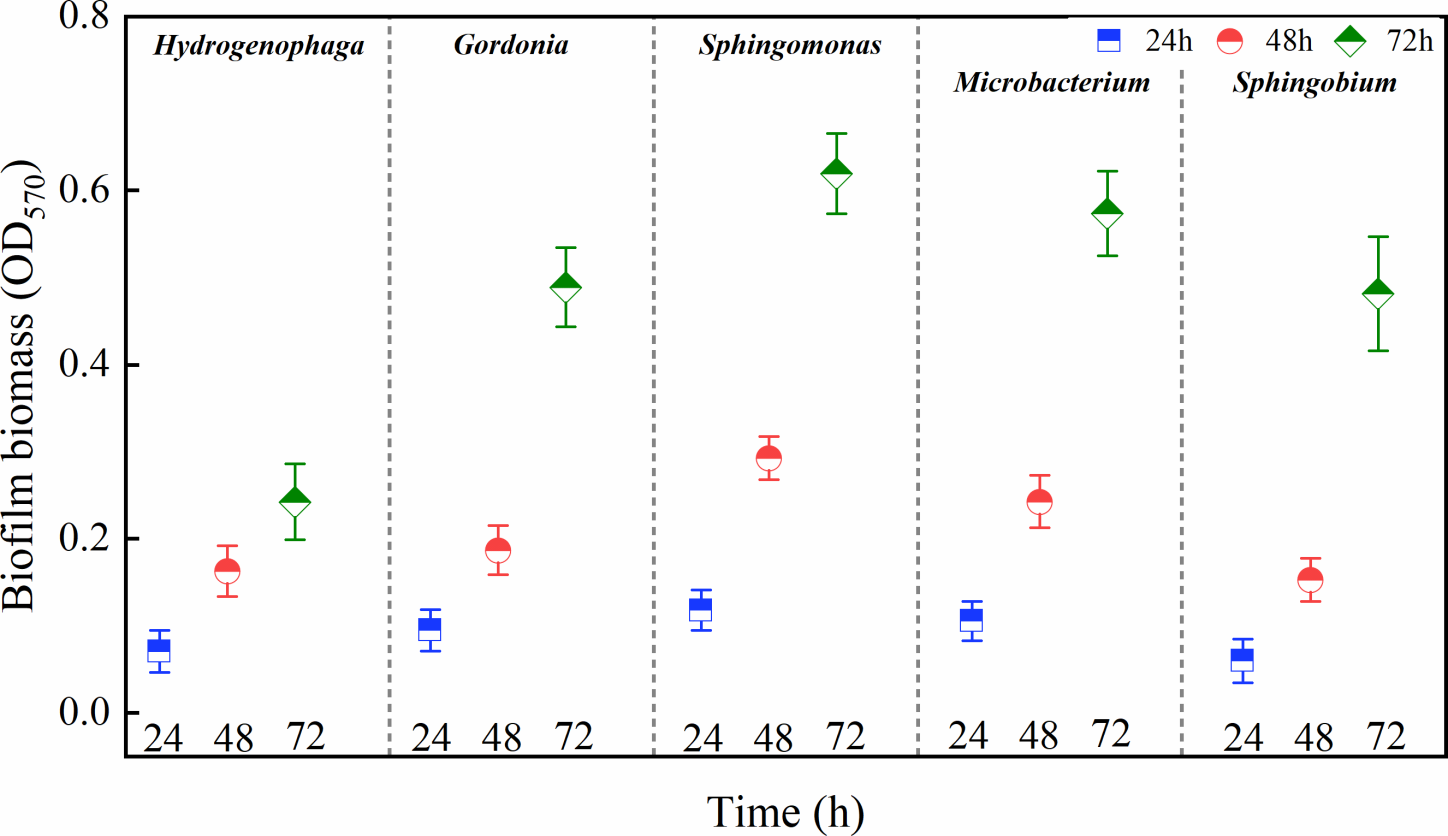
Biofilm biomass (OD_570_) of different bacteria after 24, 48, and 72 h of incubation. The data are expressed as the mean ± SEM. One-way analysis of variance (ANOVA) and a *post hoc* Tukey test were used for statistical analysis, which was based on a confidence level ≥95%.

The biofilm biomasses of the five isolated strains after 24, 48, and 72 h of incubation are depicted in Fig. 3. The biomasses gradually increased with incubation time. The highest biomass was observed at 72 h (*P* < 0.05), except for *Hyd*, whose biomass at 72 h did not differ significantly from that at 48 h (*P* > 0.05). After 24 h of incubation, the biomass of *Spm* was significantly higher than that of *Hyd* and *Spb* (*P* < 0.05), and after 48 h, it was significantly higher than that of all the other bacteria (*P* < 0.05) except for *Mic*. At 72 h, the biomass of the four strains was significantly higher than that of *Hyd* (*P* < 0.05), while no significant differences were observed among these four strains. This pattern may be attributed to their faster growth rates and higher extracellular polysaccharide secretion compared to *Hyd* (49, 50).

### Changes in the specific respiratory activity (SRA) of bacterial biofilms

**Fig 4:**
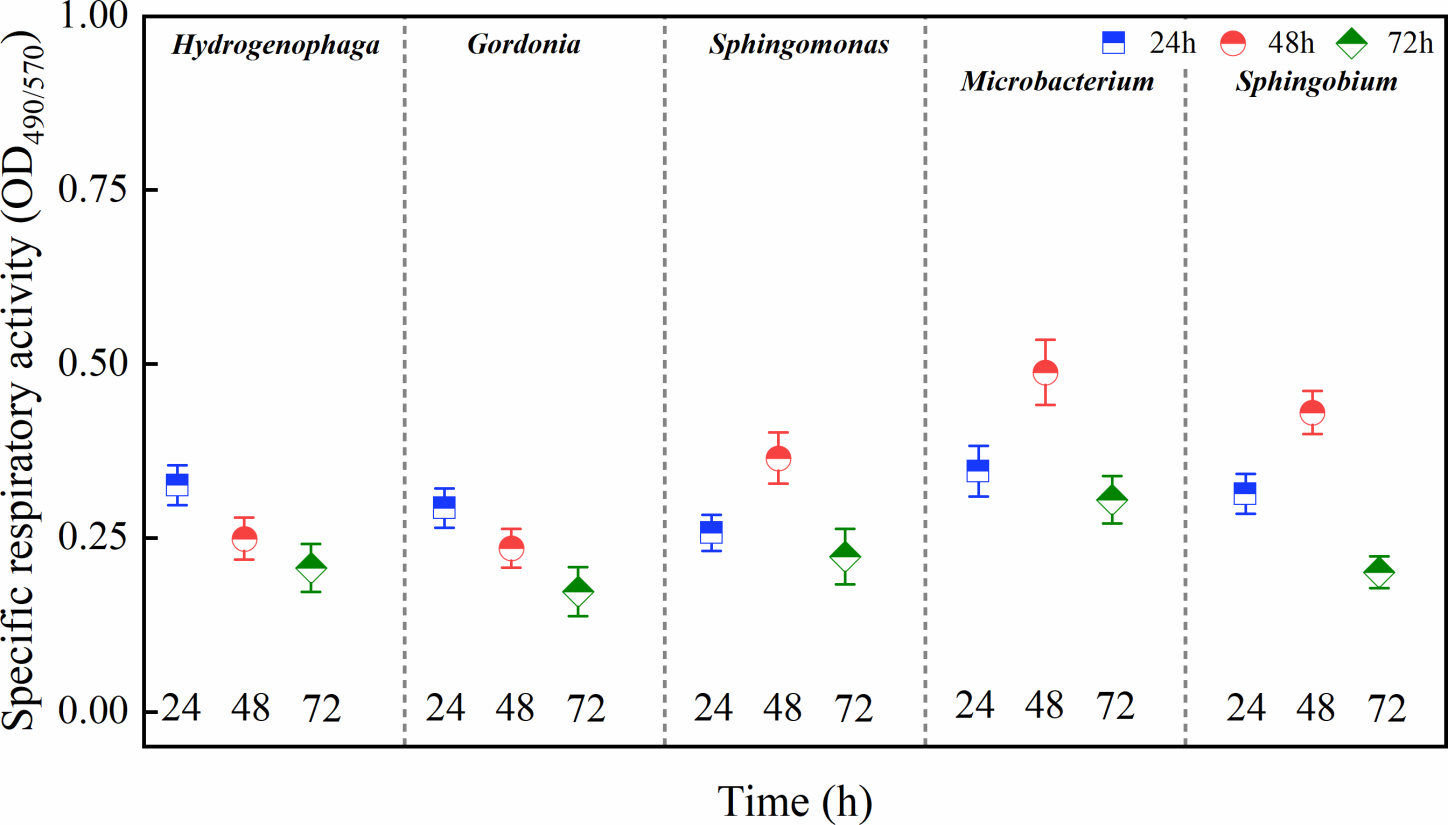
Specific respiratory activity (OD_490/570_) of the bacterial biofilms after 24, 48, and 72 h of incubation. The data are expressed as the mean ± SEM. One-way ANOVA and a *post hoc* Tukey test were used for statistical analysis, which was based on a confidence level ≥95%.

The SRAs of the biofilms at 24, 48, and 72 h are shown in Fig. 4. After 24 h of incubation, no significant differences in SRA were detected among the five strains (*P* > 0.05), and at 48 h, the SRAs of *Mic*, *Spm*, and *Spb* were significantly higher than those of *Hyd* and *Gor* (*P* < 0.05). Interestingly, no significant differences in SRA were again found among the five strains after 72 h of incubation (*P* > 0.05). Additionally, as for the trends of different strains, the SRAs of *Hyd* and *Gor* decreased with incubation time, and those of *Spm*, *Mic*, and *Spb* increased at first and then decreased.

### EPS components of the bacterial biofilms

**Fig 5:**
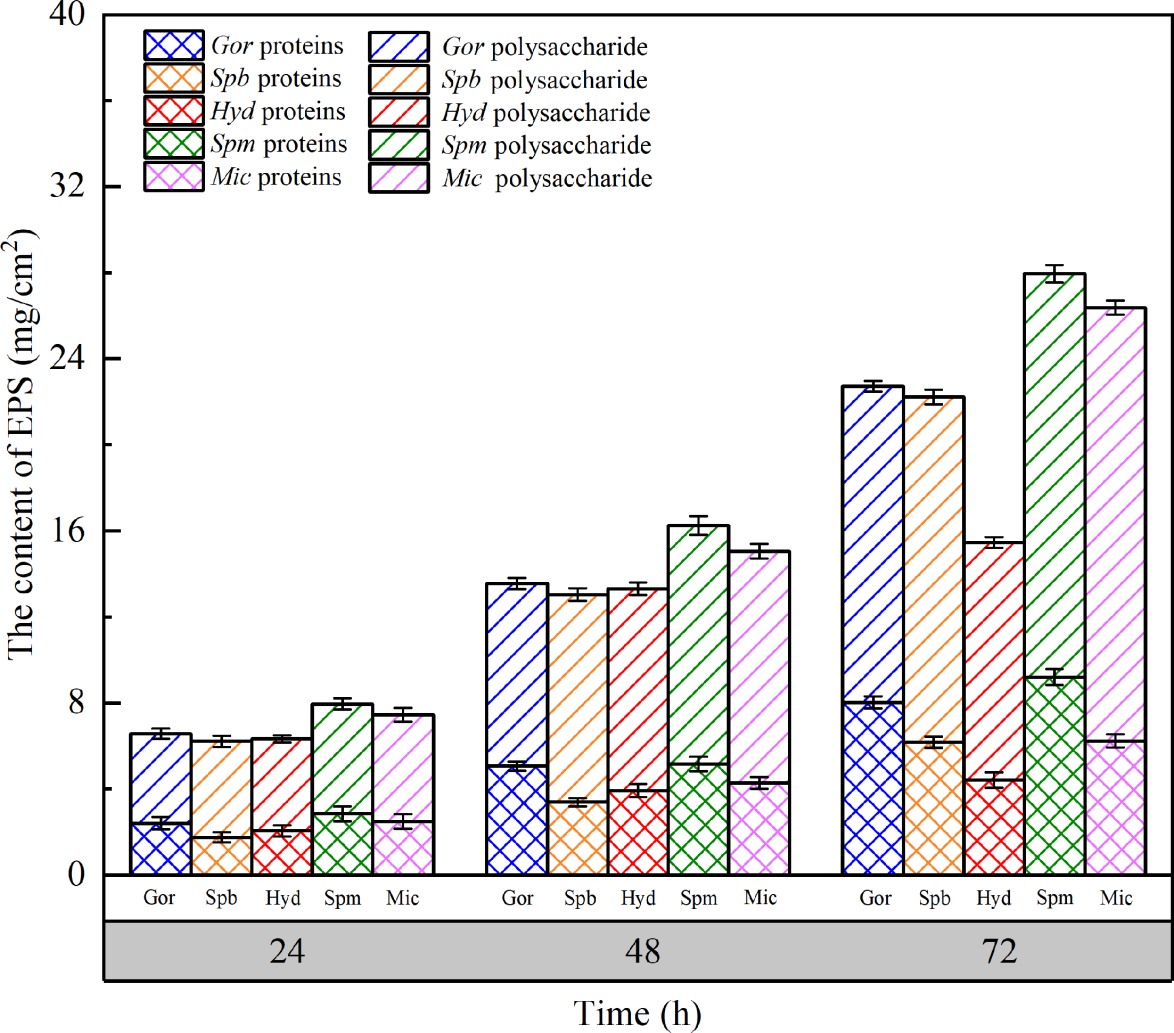
EPS content of the biofilms produced by the five strains after 24, 48, and 72 h of incubation. Data are expressed as the mean ± SEM. *Gor*, *Gordonia amicalis*; *Spb*, *Sphingobium amiense*; *Hyd*, *Hydrogenophaga laconesensis*; *Spm*, *Sphingomonas ursincola*; *Mic*, *Microbacterium saccharophilum*.

In all five strains, the contents of extracellular polysaccharides were much higher than those of extracellular proteins during incubation (Fig. 5) and accounted for 60%–70% of the biofilm EPSs.

### Comparison of BFAs

The BFA of the strains at different incubation times was determined and classified as absent, weak, medium, or strong (32). As shown in Table 1, the BFA of *Hyd* was weak, while that of *Gor* and *Mic* was moderate. *Spm* showed a strong BFA at 48 h, but this was reduced to the moderate category at 24 and 72 h. The BFA of *Spb* was weak at the beginning of incubation and became moderate at 72 h. Bacterial aggregation is an important step in biofilm formation.

**TABLE 1:** Biofilm formation ability of the five strains after 24, 48, and 72 h of incubation^*a*^

Bacteria	Biofilm forming ability after incubation time of:		
	24 h	48 h	72 h
*Hydrogenophaga laconesensis*	+	+	+
*Gordonia amicalis*	++	++	++
*Sphingomonas ursincola*	++	+++	++
*Microbacterium saccharophilum*	++	++	++
*Sphingobium amiense*	+	+	++

^*a*^ Biofilm forming ability is indicated as follows: 0, absent; +, weak; ++, medium; +++, strong.

### Bacterial AHLs

**Fig 6:**
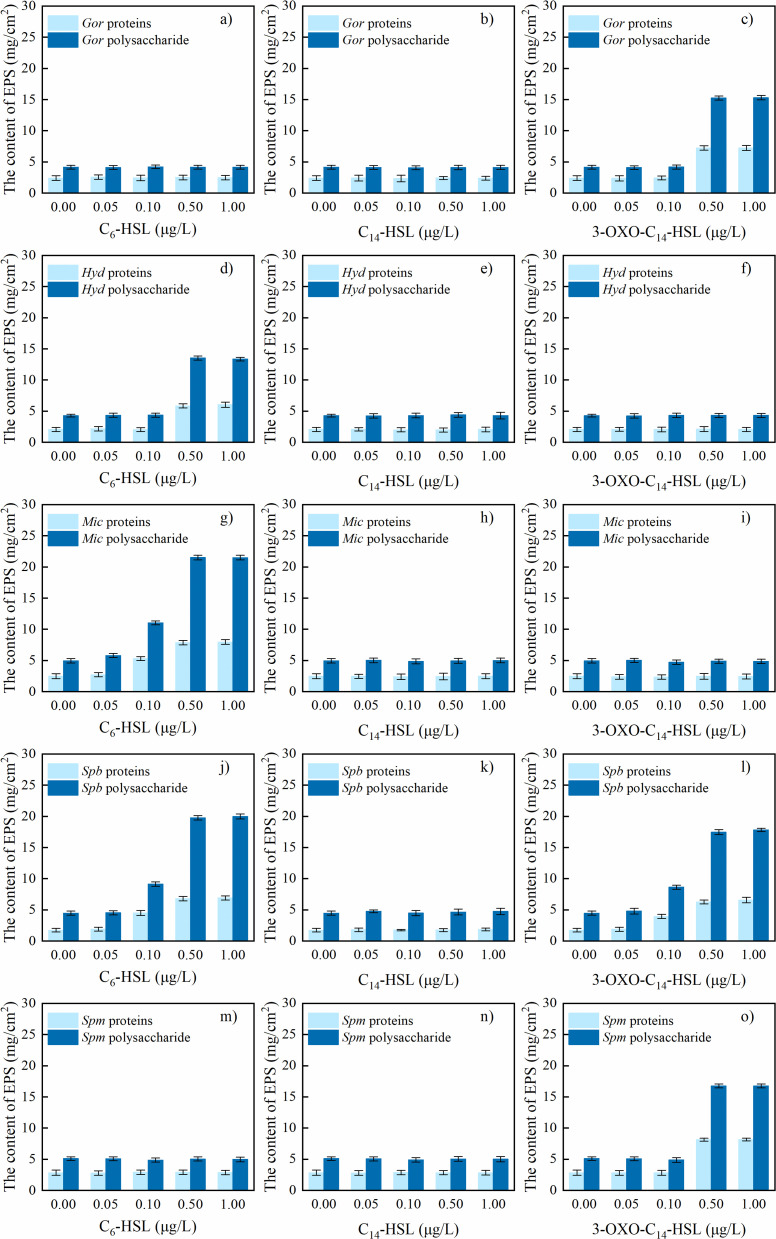
EPS content of the biofilms produced by the five strains after 24 h of incubation with different AHLs. Data are expressed as the mean ± SEM. Biofilms of (**a–c**) *Gor*, (**d–f**) *Hyd*, (**g–i**) *Mic*, (**j–l**) *Spb*, and (**m–o**) *Spm* treated with different AHLs: C6-HSL, C14-HSL, and 3-oxo-C14-HSL, respectively. *Gor*, *Gordonia amicalis*; *Spb*, *Sphingobium amiense*; *Hyd*, *Hydrogenophaga laconesensis*; *Spm*, *Sphingomonas ursincola*; *Mic*, *Microbacterium saccharophilum*.

The AHL concentrations in the five identified bacterial strains are determined in Table 2. The results showed that three of the five strains detected different AHLs during biofilm formation. Among them, C_6_-HSL, C_14_-HSL, and 3-OXO-C_14_-HSL were highly abundant, which implies that they were likely the key AHLs affecting bacterial biofilm formation. As shown in Fig. 6, supplementation with these three AHLs at varying concentrations during 24 h biofilm cultivation of the five bacterial strains revealed distinct effects on EPS production. EPS production was primarily regulated by one or two specific signaling molecules per bacterial strain. C_14_-HSL, however, had no stimulatory effect on EPS production in any of the five biofilms. In contrast, C_6_-HSL significantly enhanced EPS production in *Spb*, *Mic*, and *Hyd* biofilms. Specifically, for *Hyd*, supplementation at 0.50 μg/L significantly increased the content of both polysaccharides and proteins. *Spb* and *Mic* exhibited significantly elevated EPS content at a lower concentration of 0.10 μg/L. Similarly, 3-OXO-C_14_-HSL stimulated EPS production in *Spb*, *Gor*, and *Spm* biofilms, though the effective concentrations varied. Significant increases in EPS content for *Gor* and *Spm* were only observed at 0.50 μg/L, whereas *Spb* showed a notable enhancement in EPS content at 0.10 μg/L. These results suggest that the magnitude of EPS secretion was determined by threshold concentrations of strain-specific signaling molecules, typically comprising one or two key AHLs.

**TABLE 2:** AHL contents secreted by five bacteria

AHL	AHL content (μg/L) in:				
	*Hyd*	*Gor*	*Spm*	*Mic*	*Spb*
C_4_-HSL	ND^*a*^	ND	ND	ND	ND
C_6_-HSL	ND	ND	ND	0.2308	0.2481
C_8_-HSL	ND	ND	ND	ND	0.6392
C_10_-HSL	ND	ND	ND	ND	ND
C_12_-HSL	ND	ND	ND	ND	ND
C_14_-HSL	0.3124	ND	ND	0.3334	0.3872
3-OXO-C_6_-HSL	ND	ND	ND	ND	ND
3-OXO-C_8_-HSL	ND	ND	ND	ND	ND
3-OXO-C_10_-HSL	ND	ND	ND	ND	ND
3-OXO-C_12_-HSL	ND	ND	ND	ND	ND
3-OXO-C_14_-HSL	ND	ND	ND	ND	0.2240

^*a*^ ND indicates not detected.

### Effects of chlorine on biofilm biomass

**Fig 7:**
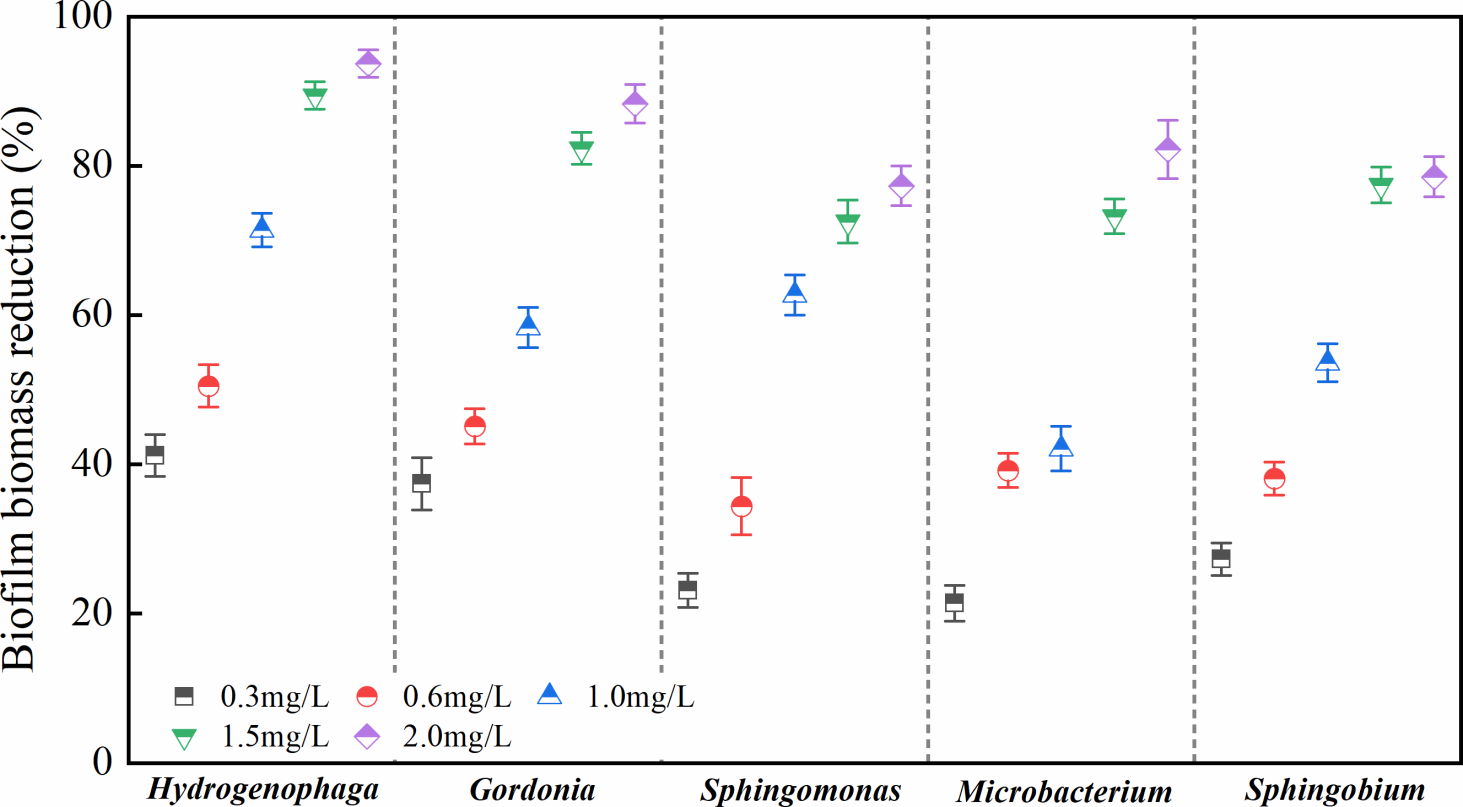
Reduction in biofilm biomass at different concentrations of residual chlorine. Data on biofilm biomass reduction at different concentrations of residual chlorine were compared using one-way ANOVA and a *post hoc* Tukey test.

The reductions in biofilm biomass after disinfection are reported in Fig. 7. At a chlorine concentration of 0.3 mg/L, the reductions in biomass did not differ significantly among *Mic*, *Spm*, and *Spb* (*P* > 0.05), but all these reductions were significantly smaller than those observed for *Hyd* and *Gor* (*P* < 0.05), which indicated that the two latter strains were more sensitive to the 0.3 mg/L dosage. The sensitivities of the five strains to 0.6 mg/L chlorine were similar to those observed at 0.3 mg/L. As the chlorine concentrations increased, the five strains showed different levels of resistance. At 1.0 mg/L, the reduction in the biomass of *Mic* was significantly smaller than that of the other four strains (*P* < 0.05). These four strains had significant differences in the reduction of residual chlorine concentration at lower residual chlorine concentration (*P* < 0.05) and were sensitive to 1.0 mg/L. For all the strains, the reduction in biomass was significantly larger at high residual chlorine concentrations (>1.0 mg/L), especially at 1.5 mg/L, than at low concentrations (*P* < 0.05). At 1.5 mg/L, there was no significant difference in biofilm reduction among *Spm*, *Mic*, and *Spb* (*P* > 0.05); however, the reduction in these three strains was significantly lower than that observed in *Hyd* and *Gor*. Results revealed that *Spm*, *Mic*, and *Spb* exhibited a stronger resistance to chlorine than *Hyd* and *Gor*. However, the reductions in biomass observed for *Spm* and *Spb* at 2.0 mg/L did not differ significantly from those at 1.5 mg/L (*P* > 0.05), but there was a significant difference in the reduction of the other three strains (*P* < 0.05). These results prove that *Spm* and *Spb* were less sensitive and more resistant to residual chlorine at concentrations ranging from 1.5 to 2.0 mg/L than the other strains.

### Effects of chlorine on the bacterial inactivation rate

**Fig 8:**
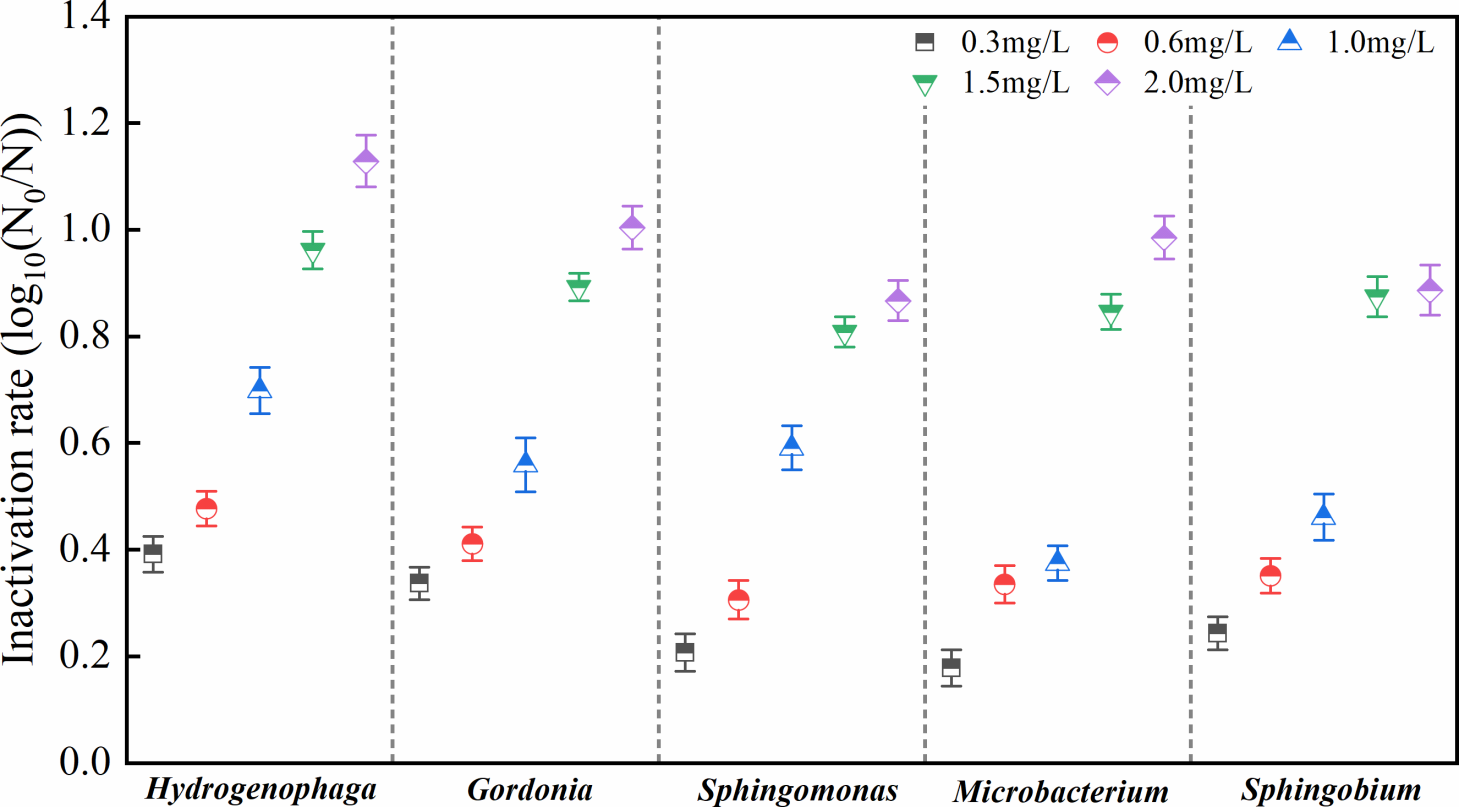
Inactivation rates of the biofilms produced by the five strains at different chlorine concentrations. One-way ANOVA and a *post hoc* Tukey test were used for statistical analysis, which was based on a confidence level ≥95%.

The bacterial inactivation rate is calculated as the logarithmic ratio of the number of bacteria before and after chlorination (51, 52). Bacteria were classified as live, damaged, or dead using flow cytometry based on the degree of damage following chlorine exposure. The bacteriostatic concentration of chlorine was then determined. The inactivation rates for the five strains under different residual chlorine concentrations (Fig. 8) and the reduction in biofilm biomass (Fig. 7) varied in a similar manner. *Mic* was not sensitive to low chlorine concentrations (≤1.0 mg/L). At 1.5 mg/L, the inactivation rates of the five bacteria significantly increased, and at 2.0 mg/L, their increment in inactivation rates decreased. Additionally, for all five strains, the proportion of damaged cells increased at first and then decreased under different residual chlorine concentrations (Tables S5 and S6). Except for the damaged *Spb* cells, which began to decrease from 33.19% at a chlorine concentration of 1.0 mg/L, the other bacteria began to decrease when the residual chlorine concentration was 0.6 mg/L. At higher concentrations ranging from 1.5 to 2.0 mg/L, the numbers of living and damaged bacteria decreased, but the reductions were not as large as those observed at 1.0–1.5 mg/L.

**Fig 9:**
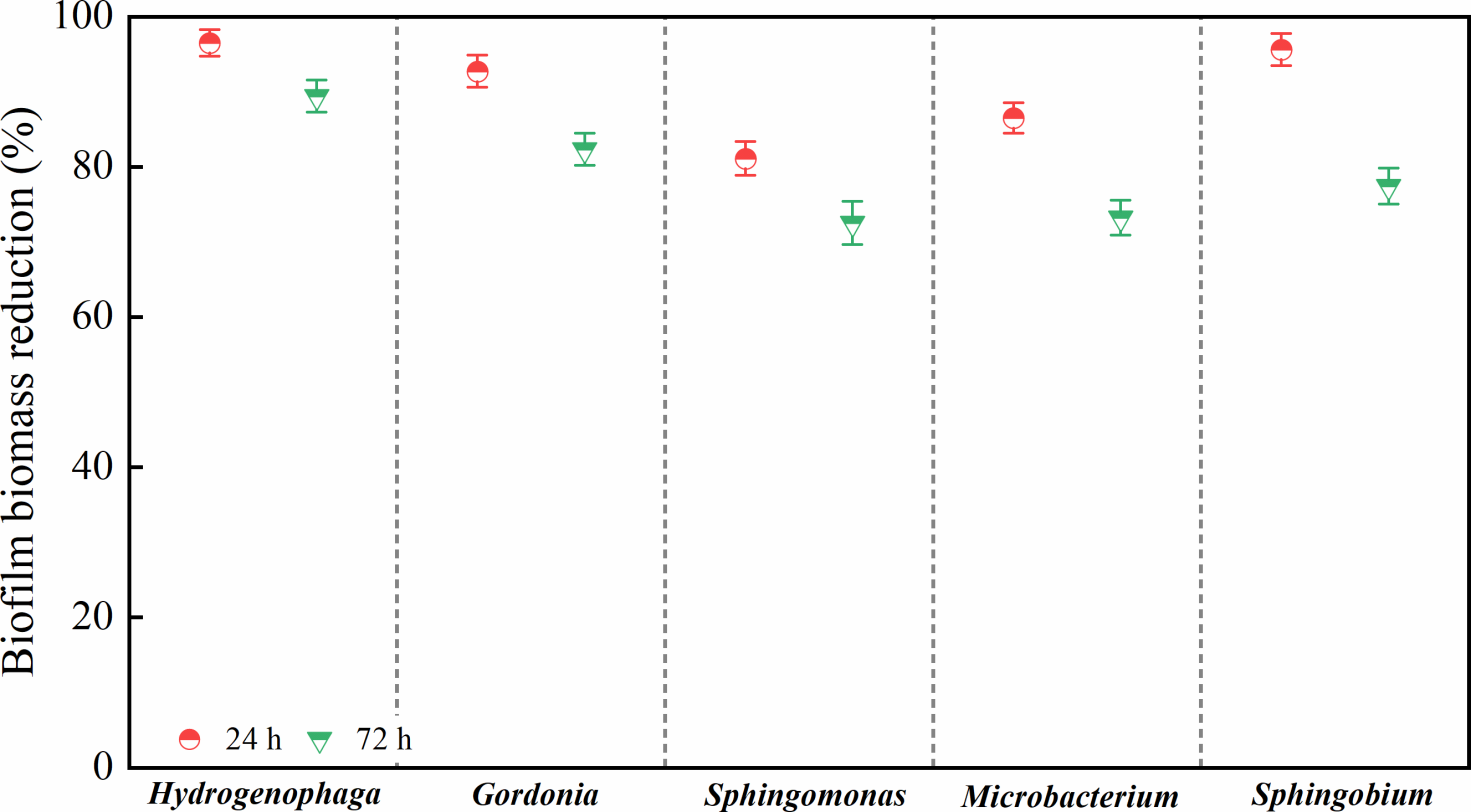
The biomass reduction at a residual chlorine concentration of 1.5 mg/L changed after 24 h and 72 h of biofilm incubation of the five strains. One-way ANOVA and a *post hoc* Tukey test were used for statistical analysis, which was based on a confidence level ≥95%.

The chlorine resistance levels of the five strains at low residual chlorine concentrations (≤1.0 mg/L) were ranked as follows: *Mic* > *Spb* > Gor > *Spm* > *Hyd. Spm* and *Hyd* were more sensitive to chlorine, whereas *Mic* was less sensitive and more resistant to it. As chlorine concentrations increased to >1.0 mg/L, the resistance levels varied, with the five strains ranked as follows: *Spm* > *Spb* > Gor > *Mic* > *Hyd. Mic* and *Hyd* were now more sensitive to chlorine, whereas *Spm*, *Spb*, and *Gor* were more resistant to it. Given the significant inhibitory effects observed at a residual chlorine concentration of 1.5 mg/L during the resistance screening of the five strains, this concentration was chosen as the threshold to investigate potential correlations between EPS content and chlorine tolerance. As shown in Fig. 9, we compared the biomass reduction of bacterial biofilms after exposure to 1.5 mg/L residual chlorine, with incubation periods of 24 and 72 h. Notably, biofilm biomass reduction decreased with longer incubation time. Moreover, all five strains exhibited significantly greater biomass reduction at 24 h compared to 72 h (*P* < 0.05). The correlation analysis demonstrated a significant negative relationship between EPS content and biomass reduction (*r* = −0.826, *P* < 0.05).

In parallel, bacterial viability post-chlorination was assessed. As indicated in Table S7, the proportion of viable cells in 24 h biofilms decreased from initial values of 77.18%–80.48% to 7.41%–11.18% after chlorine treatment, while the proportion of injured cells ranged from 6.50%–13.52%. In contrast, 72 h biofilms had substantially higher bacterial densities. Following the same chlorine treatment, the viability of cells in 72 h biofilms dropped from 78.13%–87.28% to 9.08%–12.15%, with injured cells comprising 6.95%–16.24%. The main reason for this variation in chlorine resistance was probably that the EPS content produced by the five bacteria at 24 h was less than that produced at 72 h, which protects cells from adverse environmental effects (15, 53–55). EPSs also have an effect on the biofilm structure, as they reduce the interstitial area between adjacent cells, resulting in a decrease in the surface area-to-volume ratio as well as in a reduced diffusion distance, thus hindering the transport of disinfectants to the biofilm (56).

## DISCUSSION

These isolated strains have been documented across various water supply systems, underscoring their relevance to pipeline microbial communities. Notably, the chlorine-resistant isolate *Spb* has been detected in both water treatment infrastructure and tap water samples (57, 58). Similarly, *Spm*, another chlorine-tolerant strain, has been reported not only in treatment facilities and tap water but also within DWDSs (58, 59). *Hyd* has been identified in DWDSs and biofilm reactors (60, 61). *Mic* is widely documented as a persistent microbial colonizer in distribution pipelines (60, 62, 63). Moreover, *Gor* has been significantly detected within DWDSs (64). These findings highlighted that the five strains isolated in this study were representative of the biofilm microbial populations commonly found in drinking water systems. Three of the five species, that is, *Hyd, Spb*, and *Spm,* are gram-negative bacteria. It has been reported that gram-negative bacteria are more capable of resisting disinfectants than gram-positive bacteria due to their cell wall structure (12).

Morphology was related to the ability of nutrient absorption of microorganisms. Generally, the larger the specific surface area of microorganisms, the easier it is for them to absorb nutrients. Some nutrients, such as C, N, and P, have a significant impact on the growth of microorganisms within pipelines (65). Therefore, it can be seen that most of the microorganisms in the pipeline biofilm obtained from the DWDS were small-sized bacteria. Besides, by measuring biofilm biomass and SRA of different strains respectively, it was found that the SRA of all five strains was negatively correlated with biofilm biomass. Similar results have been reported in Simoes et al. (34). This phenomenon was likely due to the biofilms being gradually formed after the bacterial cells adhered to the surface of the pipe wall, which led to the increased production of complex EPSs and consequent increase in non-metabolically active biomass, until the SRA of the biofilms eventually decreased (66, 67).

Extracellular polysaccharides assisted the bacteria in adsorption, aggregation, and biofilm formation (50), and the results of polysaccharide amounts were consistent with previous results of biofilm biomass and activities. Furthermore, among the five biofilms, that produced by *Hyd* at 72 h had the lowest EPS content. As shown in Fig. 2, the growth curve of *Hyd* was nearly stable before and after 72 h; thus, it was considered that the biofilm reached the steady state. It is understood that the bacteria use EPSs as a source of energy (68), which may explain why *Hyd* exhibited the lowest EPS content at 72 h. The weak BFA of bacteria may affect EPS contents. Ramalingam et al. (69) observed the phenomenon of self-aggregation in a *Sphingobium* sp. isolated from drinking water and, by comparing its BFA with biomass and EPS production, found that the stronger the BFA, the larger the biofilm biomass and the higher the EPS content. The variation in EPS contents during the incubation period was consistent with the decreasing SRAs of biofilms, as shown in Fig. 4. Furthermore, EPS not only enhance bacterial adhesion and promote the structural development of the biofilm but also act as a barrier. Polysaccharides, which are one of the components of EPSs, play important roles with regard to resistance to osmotic stress, antibiotics, drying, and toxic compounds (15).

As signal molecules, AHLs are believed to be used as means of communication by bacteria. When their concentration exceeds a specific threshold, bacteria will react accordingly, for example, via bioluminescence, biofilm synthesis, and other reactions (70–72). Li et al. (73) compared the biofilms of *Streptococcus mutans* NG8 and several gene deletion mutants and found a strong correlation between the ability to secrete AHLs and the ability to form biofilm. Generally, the BFA of bacteria is related to the amount of produced AHLs. However, in this study, although *Hyd* had a weak BFA and lower EPS content, its AHL production was as high as that of the other bacteria. It is possible that although *Hyd* could secrete signal molecules, their content did not reach the threshold that would allow this strain to accelerate its growth. Xia et al. (74) found that the rate of biofilm formation for *Pseudomonas aeruginosa* was accelerated and its BFA was enhanced when the 3-OXO-C_8_-HSL concentration exceeded 0.1 μg/mL.

Chlorine was generally considered to be effective in controlling biofilm formation. Among the five strains, *Hyd* exhibited the largest decrease in biofilm biomass, whereas *Mic* showed strong chlorine resistance at residual chlorine concentrations of <1.0 mg/L. For all strains, significant reductions in biomass were observed under chlorine concentrations ranging between 1.0 and 1.5 mg/L. As concentrations increased to 1.5–2.0 mg/L, the effectiveness of chlorine in removing biofilm biomass was weakened. Specifically, the two chlorine-resistant bacteria, that is, *Spm* and *Spb*, showed stronger levels of resistance to this disinfectant compared with the other strains.

In reality, the concentration of residual chlorine in China’s DWDSs is generally kept at 0.6 mg/L, and this value is even lower in water within the plumbing system. In this study, at a disinfectant concentration of 0.6 mg/L, the removal of five bacterial species ranged from 0.306 to 0.477 log. In contrast, at higher concentrations (1.0–1.5 mg/L), the reduction approached 1 log. A previous study by Oliveira et al. (75) on drinking water biofilms found that NaDCC, TCCA, and OXONE achieved less than a 1 log reduction in total cell numbers of 48 h biofilms formed on PVC and stainless-steel surfaces under all tested conditions. This level of reduction was described as “modest biofilm removal,” indicating limited effectiveness in eliminating biofilms. Further studies using similar low disinfectant doses (0.6 mg/L) have shown that such conditions still allow for the reactivation or regrowth of chlorine-tolerant bacteria, with effective suppression of reactivation typically requiring higher doses (25, 76, 77). Moreover, research on prolonged low-dose chlorination and quorum-sensing–mediated stress responses indicated that residuals below the 2–4 mg/L can induce a viable but non-culturable state in bacteria, followed by subsequent resuscitation (78, 79). Investigations in distribution systems have similarly indicated that such low residuals are more likely to maintain, rather than achieve, substantial and durable microbial reductions (24). Therefore, the reduction observed at 0.6 mg/L in this study should be considered insufficient for reliably controlling the biofilm formation of the tested isolates. In contrast, increasing the residual to 1.0–1.5 mg/L resulted in significantly higher inactivation across all isolates.

### Conclusion

**Fig 10:**
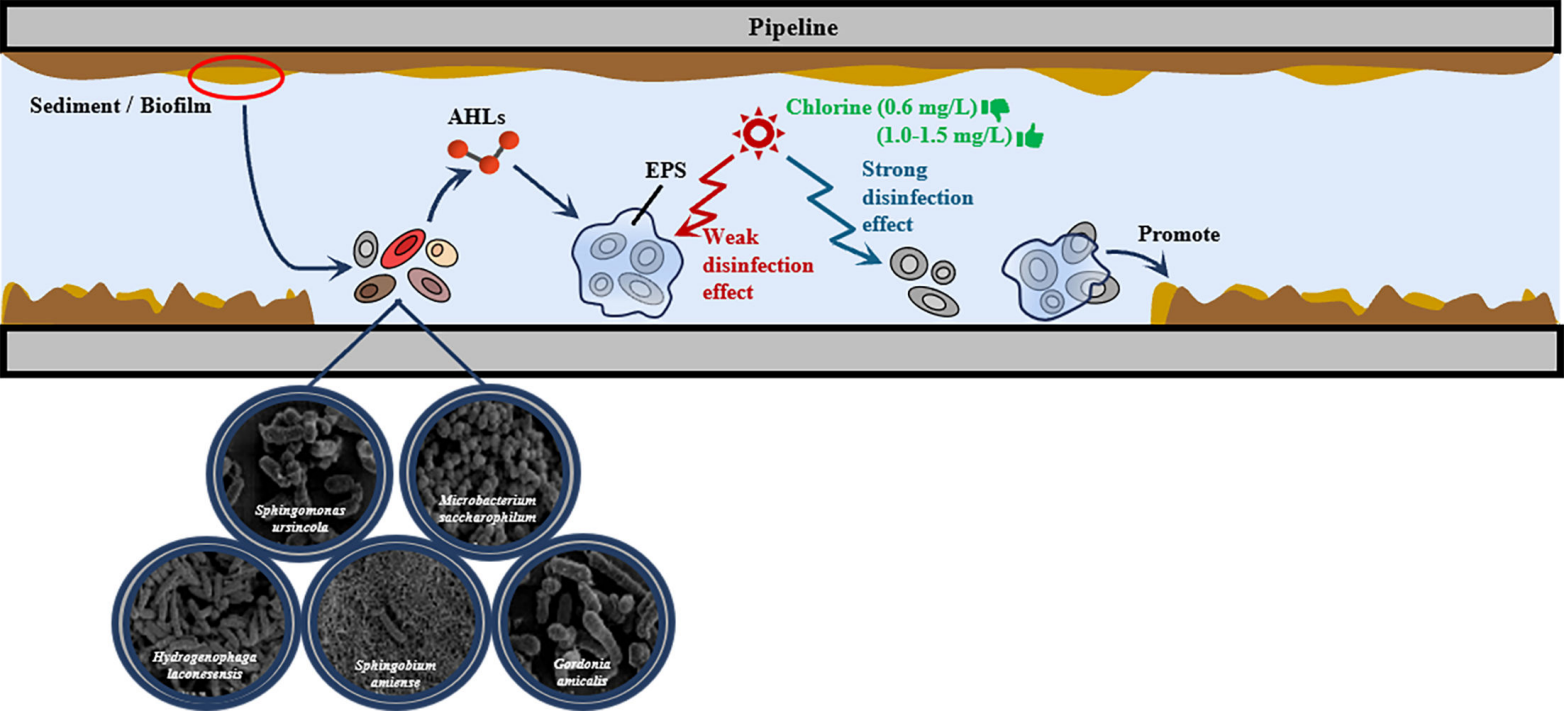
Schematic diagram of biofilm regulation and chlorine resistance of different bacterial species.

The effects of signal molecules and EPSs on biofilm formation and chlorine resistance are summarized in Fig. 10. Five strains were isolated from biofilm samples collected at a typical municipal DWDS of China and were shown to exhibit different BFAs. Among them, *S. ursincola*, *M. saccharophilum*, and *G. amicalis* had a stronger BFA and could form biofilm more rapidly. Conversely, *S. amiense*, a chlorine-resistant strain, exhibited a strong BFA only after 72 h of incubation. *H. laconesensis* showed a weak BFA. The ability to form biofilm was related to its biomass and EPS content. The production of EPSs was shown to lead to a decrease in the biofilm’s specific growth activity (OD_490/570_). Furthermore, QS played a regulatory role in these processes, utilizing AHLs as signaling molecules to mediate interbacterial communication and coordinate biofilm development. Moreover, among the detected AHLs, C_6_-HSL and 3-OXO-C_14_-HSL were present at high concentrations, which indicated that they were the main molecules in biofilm formation. In addition, this study found that biofilm EPSs could enhance bacterial resistance to chlorine disinfection. At both lower and higher residual chlorine concentrations, the resistance of *H. laconesensis* was the weakest. *M. saccharophilum* was less sensitive to chlorine at concentrations <1.0 mg/L. The strongest inhibitory effect of residual chlorine on the five strains was observed at concentrations ranging between 1.0 and 1.5 mg/L. Specifically, the two chlorine-resistant bacteria, *S. ursincola* and *S. amiense*, showed strong resistance to this disinfectant. Additionally, the numbers of damaged bacteria after disinfection should be considered, as this may cause health problems. As a large proportion of damaged bacteria was observed at a residual chlorine concentration of 0.6 mg/L, which is the level commonly maintained in DWDSs in reality, it would be recommended to increase the dosage to 1.0–1.5 mg/L to effectively control biofilm formation in the pipelines.

## Data Availability

All data supporting the results of this study are available within the paper and its supplemental material. The sequencing data for 16S rDNA have been deposited in the National Center for Biotechnology Information (NCBI) under the accession numbers PP737887 to PP737891.

## SUPPLEMENTAL MATERIAL

Supplemental material aem.01531-25-s0001.docx Tables S1 to S7 and gene sequences.

ASM does not own the copyrights to Supplemental Material that may be linked to, or accessed through, an article. The authors have granted ASM a non-exclusive, world-wide license to publish the Supplemental Material files. Please contact the corresponding author directly for reuse.
