# Supplementary material for: Species-specific chlorine resistance and biofilm regulation by extracellular polymeric substances and quorum sensing in drinking water pipeline bacteria
Source: Appl Environ Microbiol. 2026 Mar 13;92(4):e01531-25. doi: 10.1128/aem.01531-25 (PMC13101505; doi:10.1128/aem.01531-25)
Supplement: Supplemental material — Tables S1 to S7 and gene sequences. [file aem.01531-25-s0001.docx]

Supplementary Information

Table S1 Information of pipelines and water quality

| Pipe material | Pipe age (Year) | Pipe diameter (mm) | pH | DO  (mg/L) | Residual chlorine (mg/L) | Temperature  (℃) | Turbidity (NTU) | TOC  (mg/L) | NH_3_-N  (mg/L) | $\text{NO}_{\text{2}}^{\text{-}}\text{-N}$  (mg/L) | $\text{NO}_{3}^{\text{-}}\text{-N}$  (mg/L) |
| --- | --- | --- | --- | --- | --- | --- | --- | --- | --- | --- | --- |
| Gray cast iron pipe | 10 | 150 | 7.28±0.15 | 9.63±0.29 | 0.18±0.13 | 15.27±0.87 | 0.08±0.03 | 1.34±0.10 | <LOD | <LOD | 1.00±0.08 |
| Ductile iron pipe | 12 | 150 | 7.35±0.12 | 9.80±0.29 | 0.16±0.04 | 15.40±0.88 | 0.07±0.01 | 1.34±0.08 | <LOD | <LOD | 1.00±0.14 |

Note: The simulated DWDS is located at the end of the outlet of a drinking water treatment plant in a city in southern China. The water treatment process of drinking water treatment plant is a conventional treatment process, which is coagulation, precipitation, filtration and disinfection.

Table S2 HSL gradient elution condition

|  | Time (min) | A (%) | B (%) | Flow (mL/min) |
| --- | --- | --- | --- | --- |
| 1 | 2.00 min | 40.00 % | 60.00 % | 0.300 mL/min |
| 2 | 5.00 min | 0.00 % | 100.00 % | 0.300 mL/min |
| 3 | 7.00 min | 0.00 % | 100.00 % | 0.300 mL/min |
| 4 | 7.10 min | 50.00 % | 50.00 % | 0.300 mL/min |
| 5 | 10.00 min | 50.00 % | 50.00 % | 0.300 mL/min |

Table S3 Optimized Mass Spectrum parameters in MRM Mode

| Compound | Formular | Parent ion  (m/z) | Daughter ion  (m/z) | fragmentor  (V) | Collision  Energy  (V) |
| --- | --- | --- | --- | --- | --- |
| C4-HSL | C_8_H_13_NO_3_ | 172.1 | 102.1* | 70 | 8 |
| C4-HSL | C_8_H_13_NO_3_ | 172.1 | 70.6 | 70 | 8 |
| C6-HSL | C_10_H_17_NO_3_ | 200.2 | 102.1* | 60 | 8 |
| C6-HSL | C_10_H_17_NO_3_ | 200.2 | 98.5 | 60 | 10 |
| C8-HSL | C_12_H_21_NO_3_ | 228.2 | 126.6 | 80 | 10 |
| C8-HSL | C_12_H_21_NO_3_ | 228.2 | 102.1* | 80 | 8 |
| C10-HSL | C_14_H_25_NO_3_ | 256.2 | 155.0* | 90 | 10 |
| C10-HSL | C_14_H_25_NO_3_ | 256.2 | 102.1 | 90 | 8 |
| C12-HSL | C_16_H_29_NO_3_ | 284.3 | 183.1 | 150 | 10 |
| C12-HSL | C_16_H_29_NO_3_ | 284.3 | 102.1* | 150 | 8 |
| C14-HSL | C_18_H_33_NO_3_ | 312.3 | 211.2 | 120 | 10 |
| C14-HSL | C_18_H_33_NO_3_ | 312.3 | 102.1* | 120 | 8 |
| 3-OXO-C6-HSL | C_10_H_15_NO_4_ | 214.2 | 112.7 | 70 | 10 |
| 3-OXO-C6-HSL | C_10_H_15_NO_4_ | 214.2 | 102.1* | 70 | 8 |
| 3-OXO-C8-HSL | C_12_H_19_NO_4_ | 242.2 | 140.9 | 100 | 10 |
| 3-OXO-C8-HSL | C_12_H_19_NO_4_ | 242.2 | 102.1* | 100 | 8 |
| 3-OXO-C10-HSL | C_14_H_23_NO_4_ | 270.2 | 169.0 | 100 | 15 |
| 3-OXO-C10-HSL | C_14_H_23_NO_4_ | 270.2 | 102.1* | 100 | 8 |
| 3-OXO-C12-HSL | C_16_H_27_NO_4_ | 298.2 | 197.1* | 100 | 15 |
| 3-OXO-C12-HSL | C_16_H_27_NO_4_ | 298.2 | 102.1 | 100 | 8 |
| 3-OXO-C14-HSL | C_18_H_31_NO_4_ | 326.2 | 225.2* | 140 | 15 |
| 3-OXO-C14-HSL | C_18_H_31_NO_4_ | 326.2 | 102.1 | 140 | 8 |

Note: (*) Daughter ions are quantitation ions

Table S4 Basic information of bacteria

| Code name | Identification of bacteria species  (Per. ident) | Taxonomic status | Colony morphology | Cell morphology | | | The source of bacterial isolation in other studies | Reference |
| --- | --- | --- | --- | --- | --- | --- | --- | --- |
|  |  |  |  | Shape | Gram | Biochemical reaction |  |  |
| *Spb* | *Sphingobium amiense*  (98.74%) | Alphaproteobacteria | Round, convex, smooth, golden colony | Rod shape | G^−^ | Catalase (+)  Starch hydrolysis (–)  Gelatin hydrolysis (–)  Casein hydrolysis (+) | Water plant structure, tap water | Vaz-Moreira *et al.* [59,60] |
| *Hyd* | *Hydrogenophaga laconesensis*  (99.04%) | Betaproteobacteria | Convex, not smooth, milky colony | Rod shape | G^−^ | Catalase (+)  Starch hydrolysis (–)  Gelatin hydrolysis (–)  Casein hydrolysis (–) | Drinking water distribution system, biofilm reactor | Marcus *et al.* [62],  Zhang *et al.* [63] |
| *Spm* | *Sphingomonas ursincola*  (99.93%) | Alphaproteobacteria | Round, convex, smooth, brown colony | Rod or coccoid shape | G^−^ | Catalase (+)  Starch hydrolysis (+)  Gelatin hydrolysis (–)  Casein hydrolysis (+) | Drinking water distribution system, water plant structure, tap water | Koskinen *et al.* [61],  Vaz-Moreira *et al.* [60] |
| *Mic* | *Microbacterium saccharophilum*  (99.93%) | Actinomycetes | Round, convex, smooth, dark brown colony | Rod shape | G^+^ | Catalase (+)  Starch hydrolysis (+)  Gelatin hydrolysis (+)  Casein hydrolysis (–) | Drinking water distribution system | Marcus *et al.* [62],  Wang *et al.* [64],  Yu *et al.* [65] |
| *Gor* | *Gordonia amicalis*  (99.86%) | Actinomycetes | Convex, not smooth, orange colony | Rod shape | G^+^ | Catalase (+)  Starch hydrolysis (+)  Gelatin hydrolysis (–)  Casein hydrolysis (–) | Drinking water distribution system | Lu *et al.* [66] |

Table S5 The number of five kinds of bacteria before chlorine treatment

| Bacteria | Number of living bacteria | Number of dead bacteria | Number of damaged bacteria |
| --- | --- | --- | --- |
|  | (log_10_N±SD) | (log_10_N±SD) | (log_10_N±SD) |
| *Hydrogenophaga laconesensis* | 7.145±0.0480（83.27%） | 5.186±0.0964（1.01%） | 5.835±0.0375（4.27%） |
| *Gordonia amicalis* | 7.600±0.0126（84.99%） | 6.000±0.0195（2.25%） | 6.432±0.1516（6.35%） |
| *Sphingomonas ursincola* | 7.925±0.0020（78.13%） | 6.681±0.0165（5.08%） | 6.875±0.0438（7.48%） |
| *Microbacterium saccharophilum* | 7.866±0.0097（82.98%） | 6.787±0.0460（7.30%） | 6.421±0.0523（3.34%） |
| *Sphingobium amiense* | 7.546±0.0109（87.28%） | 5.870±0.1611（1.91%） | 6.122±0.0523（3.21%） |

Table S6 The number of five kinds of bacteria after chlorine treatment

(a)

| Bacteria | After 0.3 mg/L chlorine treatment | | |
| --- | --- | --- | --- |
|  | Number of living bacteria (log_10_N±SD) | Number of dead bacteria (log_10_N±SD) | Number of damaged bacteria (log_10_N±SD) |
| *Hydrogenophaga laconesensis* | 6.754±0.0574（33.74%） | 6.524±0.0299（21.37%） | 6.729±0.0346（33.45%） |
| *Gordonia amicalis* | 7.263±0.0356（39.17%） | 7.016±0.0137（23.31%） | 7.143±0.0233（31.11%） |
| *Sphingomonas ursincola* | 7.718±0.0164（48.50%） | 7.143±0.0098（14.75%） | 7.441±0.0197（27.44%） |
| *Microbacterium saccharophilum* | 7.688±0.0238（55.06%） | 7.269±0.0357（22.22%） | 7.122±0.0425（16.33%） |
| *Sphingobium amiense* | 7.308±0.0142（48.02%） | 6.854±0.0206（17.37%） | 7.053±0.0336（27.01%） |

(b)

| Bacteria | After 0.6 mg/L chlorine treatment | | |
| --- | --- | --- | --- |
|  | Number of living bacteria (log_10_N±SD) | Number of dead bacteria (log_10_N±SD) | Number of damaged bacteria (log_10_N±SD) |
| *Hydrogenophaga laconesensis* | 6.668±0.0401（27.69%） | 6.610±0.0542（26.06%） | 6.747±0.0343（34.80%） |
| *Gordonia amicalis* | 7.189±0.0378（33.02%） | 7.077±0.0270（26.81%） | 7.178±0.0155（33.76%） |
| *Sphingomonas ursincola* | 7.619±0.0123（38.60%） | 7.283±0.0371（20.41%） | 7.504±0.0118（31.67%） |
| *Microbacterium saccharophilum* | 7.531±0.0154（38.40%） | 7.392±0.0415（29.30%） | 7.324±0.0672（25.91%） |
| *Sphingobium amiense* | 7.199±0.0140（37.44%） | 6.955±0.0109（21.89%） | 7.141±0.0101（33.08%） |

(c)

| Bacteria | After 1.0 mg/L chlorine treatment | | |
| --- | --- | --- | --- |
|  | Number of living bacteria (log_10_N±SD) | Number of dead bacteria (log_10_N±SD) | Number of damaged bacteria (log_10_N±SD) |
| *Hydrogenophaga laconesensis* | 6.447±0.0332（16.66%） | 6.942±0.0517（56.02%） | 6.405±0.0189（15.88%） |
| *Gordonia amicalis* | 7.041±0.0027（23.48%） | 7.282±0.0471（43.02%） | 7.083±0.0159（27.09%） |
| *Sphingomonas ursincola* | 7.334±0.0129（20.04%） | 7.673±0.0132（49.93%） | 7.319±0.0176（20.71%） |
| *Microbacterium saccharophilum* | 7.492±0.0225（35.03%） | 7.529±0.0192（40.27%） | 7.173±0.0752（18.31%） |
| *Sphingobium amiense* | 7.090±0.0405（29.17%） | 7.091±0.0386（30.04%） | 6.962±0.0247（33.19%） |

(d)

| Bacteria | After 1.5 mg/L chlorine treatment | | |
| --- | --- | --- | --- |
|  | Number of living bacteria (log_10_N±SD) | Number of dead bacteria (log_10_N±SD) | Number of damaged bacteria (log_10_N±SD) |
| *Hydrogenophaga laconesensis* | 6.183±0.0633（9.08%） | 7.054±0.0420（72.53%） | 6.047±0.0262（6.95%） |
| *Gordonia amicalis* | 6.708±0.0340（10.90%） | 7.508±0.0271（72.29%） | 6.667±0.0370（10.40%） |
| *Sphingomonas ursincola* | 7.117±0.0127（12.15%） | 7.763±0.0051（61.41%） | 7.237±0.0071（17.12%） |
| *Microbacterium saccharophilum* | 7.020±0.0071（11.84%） | 7.785±0.0402（72.49%） | 6.877±0.0865（9.28%） |
| *Sphingobium amiense* | 6.676±0.0260（11.23%） | 7.427±0.0080（64.93%） | 6.832±0.0111（16.24%） |

(e)

| Bacteria | After 2.0 mg/L chlorine treatment | | |
| --- | --- | --- | --- |
|  | Number of living bacteria (log_10_N±SD) | Number of dead bacteria (log_10_N±SD) | Number of damaged bacteria (log_10_N±SD) |
| *Hydrogenophaga laconesensis* | 6.016±0.0470（6.19%） | 7.093±0.0414（79.25%） | 5.698±0.0644（3.12%） |
| *Gordonia amicalis* | 6.596±0.0263（8.42%） | 7.549±0.0322（79.47%） | 6.404±0.0221（5.70%） |
| *Sphingomonas ursincola* | 7.058±0.0188（10.62%） | 7.810±0.0077（68.48%） | 7.067±0.0132（11.58%） |
| *Microbacterium saccharophilum* | 6.881±0.0245（8.59%） | 7.814±0.0421（77.57%） | 6.783±0.0603（7.46%） |
| *Sphingobium amiense* | 6.664±0.0291（10.92%） | 7.470±0.0062（71.70%） | 6.612±0.0240（9.78%） |

Table S7 Effects of incubation time on bacterial number following chlorine disinfection across five bacteria

(a) The biofilms of five kinds of bacteria were incubated for 24 h

| Bacteria | Number of living bacteria | Number of dead bacteria | Number of damaged bacteria | After 1.5 mg/L chlorine treatment | | |
| --- | --- | --- | --- | --- | --- | --- |
|  |  |  |  | Number of living bacteria | Number of dead bacteria | Number of damaged bacteria |
|  | (log_10_N±SD) | (log_10_N±SD) | (log_10_N±SD) | (log_10_N±SD) | (log_10_N±SD) | (log_10_N±SD) |
| *Hyd* | 5.958±0.0162（80.48%） | 4.595±0.0075（3.49%） | 4.679±0.0296（4.23%） | 4.934±0.0330（7.61%） | 5.922±0.0125（74.08%） | 4.864±0.0417（6.50%） |
| *Gor* | 6.442±0.0172（77.58%） | 4.945±0.0169（2.47%） | 5.098±0.0129（3.52%） | 5.476±0.0126（8.39%） | 6.366±0.0186（65.12%） | 5.554±0.0116（10.06%） |
| *Spm* | 6.608±0.0025（77.18%） | 5.325±0.0809（4.09%） | 5.483±0.0186（5.80%） | 5.769±0.0089（11.18%） | 6.515±0.0106（62.37%） | 5.851±0.0135（13.52%） |
| *Mic* | 6.552±0.0079（79.48%） | 5.316±0.0395（4.62%） | 5.243±0.0190（3.90%） | 5.669±0.0107（10.40%） | 6.472±0.0093（66.06%） | 5.714±0.0185（11.55%） |
| *Spb* | 5.844±0.0086（79.80%） | 4.212±0.1218（1.93%） | 4.375±0.0392（2.72%） | 4.812±0.0012（7.41%） | 5.785±0.0102（69.57%） | 4.815±0.0322（7.47%） |

(b) The biofilms of five kinds of bacteria were incubated for 72 h

| Bacteria | Number of living bacteria | Number of dead bacteria | Number of damaged bacteria | After 1.5 mg/L chlorine treatment | | |
| --- | --- | --- | --- | --- | --- | --- |
|  |  |  |  | Number of living bacteria | Number of dead bacteria | Number of damaged bacteria |
|  | (log_10_N±SD) | (log_10_N±SD) | (log_10_N±SD) | (log_10_N±SD) | (log_10_N±SD) | (log_10_N±SD) |
| *Hyd* | 7.145±0.0480（83.27%） | 5.186±0.0964（1.01%） | 5.835±0.0375（4.27%） | 6.183±0.0633（9.08%） | 7.054±0.0420（72.53%） | 6.047±0.0262（6.95%） |
| *Gor* | 7.600±0.0126（84.99%） | 6.000±0.0195（2.25%） | 6.432±0.1516（6.35%） | 6.708±0.0340（10.90%） | 7.508±0.0271（72.29%） | 6.667±0.0370（10.40%） |
| *Spm* | 7.925±0.0020（78.13%） | 6.681±0.0165（5.08%） | 6.875±0.0438（7.48%） | 7.117±0.0127（12.15%） | 7.763±0.0051（61.41%） | 7.237±0.0071（17.12%） |
| *Mic* | 7.866±0.0097（82.98%） | 6.787±0.0460（7.30%） | 6.421±0.0523（3.34%） | 7.020±0.0071（11.84%） | 7.785±0.0402（72.49%） | 6.877±0.0865（9.28%） |
| *Spb* | 7.546±0.0109（87.28%） | 5.870±0.1611（1.91%） | 6.122±0.0523（3.21%） | 6.676±0.0260（11.23%） | 7.427±0.0080（64.93%） | 6.832±0.0111（16.24%） |

The bacteria full-length sequence of 16S rRNA gene

*Sphingobium amiense:*

GGCTCAGAACGAACGCTGGCGGCATGCCTAATACATGCAAGTCGAACGAGACCTTCGGGTCTAGTGGCGCACGGGTGCGTAACGCGTGGGAATCTGCCCTTGGGTTCGGAATAACATCGGGAAACTGATGCTAATACCGGATGATGACGTAAGTCCAAAGATTTATCGCCCAGGGATGAGCCCGCGTAGGATTAGCTAGTTGGTGGGGTAAAGGCCTACCAAGGCGACGATCCTTAGCTGGTCTGAGAGGATGATCAGCCACACTGGGACTGAGACACGGCCCAGACTCCTACGGGAGGCAGCAGTAGGGAATATTGGACAATGGGGGCAACCCTGATCCAGCAATGCCGCGTGAGTGATGAAGGCCTTAGGGTTGTAAAGCTCTTTTACCCGGGATGATAATGACAGTACCGGGAGAATAAGCCCCGGCTAACTCCGTGCCAGCAGCCGCGGTAATACGGAGGGGGCTAGCGTTGTTCGGAATTACTGGGCGTAAAGCGCACGTAGGCGGCGATTTAAGTCAGAGGTGAAAGCCCGGGGCTCAACCCCGGAACTGCCTTTGAGACTGGATTGCTTGAACATCGGAGAGGTGAGTGGAATTCCGAGTGTAGAGGTGAAATTCGTAGATATTCGGAAGAACACCAGTGGCGAAGGCGGCTCACTGGACGATTGTTGACGCTGAGGTGCGAAAGCGTGGGGAGCAAACAGGATTAGATACCCTGGTAGTCCACGCCGTAAACGATGATAACTAGCTGCCGGGGCACATGGTGTTTCGGTGGCGCAGCTAACGCATTAAGTTATCCGCCTGGGGAGTACGGTCGCAAGATTAAAACTCAAAGGAATTGACGGGGGCCTGCACAAGCGGTGGAGCATGTGGTTTAATTCGAAGCAACGCGCAGAACCTTACCAACGTTTGACATCCCTATCGCGGATCGTGGAGACACTTTCCTTCAGTTCGGCTGGATAGGTGACAGGTGCTGCATGGCTGTCGTCAGCTCGTGTCGTGAGATGTTGGGTTAAGTCCCGCAACGAGCGCAACCCTCGCCTTTAGTTGCCAGCATTTAGTTGGGTACTCTAAAGGAACCGCCGGTGATAAGCCGGAGGAAGGTGGGGATGACGTCAAGTCCTCATGGCCCTTACGCGTTGGGCTACACACGTGCTACAATGGCGACTACAGTGGGCAGCGACCTCGCGAGGGGGAGCTAATCTCCAAAAGTCGTCTCAGTTCGGATCGTTCTCTGCAACTCGAGAGCGTGAAGGCGGAATCGCTAGTAATCGCGGATCAGCATGCCGCGGTGAATACGTTCCCAGGCCTTGTACACACCGCCCGTCACACCATGGGAGTTGGATTCACTCGAAGGCGTTGAGCTAACCGCAAGGAGGCAGGCGACCACAGTGGGTTTAGCGACTGGGGTGAAGTCGT

*Hydrogenophaga laconesensis:*

GGCTCAGATTGAACGCTGGCGGCATGCTTTACACATGCAAGTCGAACGGTAACAGGCCGCAAGGTGCTGACGAGTGGCGAACGGGTGAGTAATGCATCGGAACGTGCCCAGTCGTGGGGGATAACGCAGCGAAAGCTGCGCTAATACCGCATACGATCTATGGATGAAAGCGGGGGACCGTAAGGCCTCGCGCGATTGGAGCGGCCGATGTCAGATTAGGTAGTTGGTGAGGTAAAGGCTCACCAAGCCAACGATCTGTAGCTGGTCTGAGAGGACGACCAGCCACACTGGGACTGAGACACGGCCCAGACTCCTACGGGAGGCAGCAGTGGGGAATTTTGGACAATGGGCGCAAGCCTGATCCAGCAATGCCGCGTGCAGGAAGAAGGCCTTCGGGTTGTAAACTGCTTTTGTACGGAACGAAACGGCTCTCACTAATACTGGGGGCTAATGACGGTACCGTAAGAATAAGCACCGGCTAACTACGTGCCAGCAGCCGCGGTAATACGTAGGGTGCAAGCGTTAATCGGAATTACTGGGCGTAAAGCGTGCGCAGGCGGTTGTGTAAGACAGGCGTGAAATCCCCGGGCTCAACCTGGGAATTGCGCTTGTGACTGCACAGCTGGAGTGCGGCAGAGGGGGATGGAATTCCGCGTGTAGCAGTGAAATGCGTAGATATGCGGAGGAACACCGATGGCGAAGGCAATCCCCTGGGCCTGCACTGACGCTCATGCACGAAAGCGTGGGGAGCAAACAGGATTAGATACCCTGGTAGTCCACGCCCTAAACGATGTCAACTGGTTGTTGGGTCTCTTCTGACTCAGTAACGAAGCTAACGCGTGAAGTTGACCGCCTGGGGAGTACGGCCGCAAGGTTGAAACTCAAAGGAATTGACGGGGACCCGCACAAGCGGTGGATGATGTGGTTTAATTCGATGCAACGCGAAAAACCTTACCCACCTTTGACATGTACGGAAGTTGCCAGAGATGGCTTCGTGCTCGAAAGAGAGCCGTAACACAGGTGCTGCATGGCTGTCGTCAGCTCGTGTCGTGAGATGTTGGGTTAAGTCCCGCAACGAGCGCAACCCTTGCCATTAGTTGCTACGAAAGGGCACTCTAATGGGACTGCCGGTGACAAACCGGAGGAAGGTGGGGATGACGTCAAGTCCTCATGGCCCTTATAGGTGGGGCTACACACGTCATACAATGGCTGGTACAAAGGGTTGCCAACCCGCGAGGGGGAGCCAATCCCATAAAGCCAGTCGTAGTCCGGATCGCAGTCTGCAACTCGACTGCGTGAAGTCGGAATCGCTAGTAATCGTGGATCAGCATGTCACGGTGAATACGTTCCCGGGTCTTGTACACACCGCCCGTCACACCATGGGAGCGGGTCTCGCCAGAAGTAGTTAGCCTAACCGCAAGGAGGGCGATTACCACGGCGGGGTTCGTGACTGGGGTGAAGTCGT

*Sphingomonas ursincola:*

GGCTCAGAACGAACGCTGGCGGCATGCCTAACACATGCAAGTCGAACGAAGGCTTCGGCCTTAGTGGCGCACGGGTGCGTAACGCGTGGGAATCTGCCCTTGGGTTCGGAATAACAGTGAGAAATTACTGCTAATACCGGATGATGACTTCGGTCCAAAGATTTATCGCCCAAGGATGAGCCCGCGTAAGATTAGCTAGTTGGTGAGGTAAAGGCTCACCAAGGCGACGATCTTTAGCTGGTCTGAGAGGATGATCAGCCACACTGGGACTGAGACACGGCCCAGACTCCTACGGGAGGCAGCAGTGGGGAATATTGGACAATGGGCGAAAGCCTGATCCAGCAATGCCGCGTGAGTGATGAAGGCCTTAGGGTTGTAAAGCTCTTTTACCAGGGATGATAATGACAGTACCTGGAGAATAAGCTCCGGCTAACTCCGTGCCAGCAGCCGCGGTAATACGGAGGGAGCTAGCGTTGTTCGGAATTACTGGGCGTAAAGCGCACGTAGGCGGCCATTCAAGTCAGAGGTGAAAGCCCGGGGCTCAACCCCGGAACTGCCTTTGAAACTAGATGGCTTGAATCTTGGAGAGGCGAGTGGAATTCCGAGTGTAGAGGTGAAATTCGTAGATATTCGGAAGAACACCAGTGGCGAAGGCGACTCGCTGGACAAGTATTGACGCTGAGGTGCGAAAGCGTGGGGAGCAAACAGGATTAGATACCCTGGTAGTCCACGCCGTAAACGATGATAACTAGCTGTCCGGGTTCATGGAACTTGGGTGGCGCAGCTAACGCATTAAGTTATCCGCCTGGGGAGTACGGTCGCAAGATTAAAACTCAAAGGAATTGACGGGGGCCTGCACAAGCGGTGGAGCATGTGGTTTAATTCGAAGCAACGCGCAGAACCTTACCAGCGTTTGACATGTCTAGTATGTTTCCCAGAGATGGGTTACTTCAGTTCGGCTGGCTAGAACACAGGTGCTGCATGGCTGTCGTCAGCTCGTGTCGTGAGATGTTGGGTTAAGTCCCGCAACGAGCGCAACCCTCGTCTTTAGTTGCCATCATTTAGTTGGGCACTCTAAAGAAACCGCCGGTGATAAGCCGGAGGAAGGTGGGGATGACGTCAAGTCCTCATGGCCCTTACACGCTGGGCTACACACGTGCTACAATGGCGGTGACAGTGAGCAGCTAGATCGCGAGATCATGCTAATCTCAAAAAGCCGTCTCAGTTCGGATTGTTCTCTGCAACTCGAGAGCATGAAGGCGGAATCGCTAGTAATCGCGGATCAGCATGCCGCGGTGAATACGTTCCCAGGCCTTGTACACACCGCCCGTCACACCATGGGAGTTGGATTCACCCGAAGGCGCTGCGCTAACCCGCAAGGGAGGCAGGCGACCACGGTGGGTTCAGCGACTGGGGTGAAGTCGT

*Microbacterium saccharophilum:*

GGCTCAGGATGAACGCTGGCGGCGTGCTTAACACATGCAAGTCGAACGGTGAAAGAGAGCTTGCTCTCTGGATCAGTGGCGAACGGGTGAGTAACACGTGAGCAACCTGCCCCGGACTCTGGGATAACAGCTGGAAACAGCTGCTAATACCGGATACGAGCTGCGAAGGCATCTTCAGCAGCTGGAAAGAATTTCGGTCCGGGATGGGCTCGCGGCCTATCAGCTAGTTGGTGAGGTAATGGCTCACCAAGGCGTCGACGGGTAGCCGGCCTGAGAGGGTGACCGGCCACACTGGGACTGAGACACGGCCCAGACTCCTACGGGAGGCAGCAGTGGGGAATATTGCACAATGGGCGAAAGCCTGATGCAGCAACGCCGCGTGAGGGATGACGGCCTTCGGGTTGTAAACCTCTTTTAGCAGGGAAGAAGCGAAAGTGACGGTACCTGCAGAAAAAGCGCCGGCTAACTACGTGCCAGCAGCCGCGGTAATACGTAGGGCGCAAGCGTTATCCGGAATTATTGGGCGTAAAGAGCTCGTAGGCGGTTTGTCGCGTCTGCTGTGAAAACCCGAGGCTCAACCTCGGGCCTGCAGTGGGTACGGGCAGACTAGAGTGCGGTAGGGGAGATTGGAATTCCTGGTGTAGCGGTGGAATGCGCAGATATCAGGAGGAACACCGATGGCGAAGGCAGATCTCTGGGCCGTAACTGACGCTGAGGAGCGAAAGGGTGGGGAGCAAACAGGCTTAGATACCCTGGTAGTCCACCCCGTAAACGTTGGGAACTAGTTGTGGGGTCCATTCCACGGATTCCGTGACGCAGCTAACGCATTAAGTTCCCCGCCTGGGGAGTACGGCCGCAAGGCTAAAACTCAAAGGAATTGACGGGGACCCGCACAAGCGGCGGAGCATGCGGATTAATTCGATGCAACGCGAAGAACCTTACCAAGGCTTGACATATACGAGAACGGGCCAGAAATGGTCAACTCTTTGGACACTCGTAAACAGGTGGTGCATGGTTGTCGTCAGCTCGTGTCGTGAGATGTTGGGTTAAGTCCCGCAACGAGCGCAACCCTCGTTCTATGTTGCCAGCACGTAATGGTGGGAACTCATGGGATACTGCCGGGGTCAACTCGGAGGAAGGTGGGGATGACGTCAAATCATCATGCCCCTTATGTCTTGGGCTTCACGCATGCTACAATGGCCGGTACAAAGGGCTGCAATACCGCAAGGTGGAGCGAATCCCAAAAAGCCGGTCCCAGTTCGGATTGAGGTCTGCAACTCGACCTCATGAAGTCGGAGTCGCTAGTAATCGCAGATCAGCAACGCTGCGGTGAATACGTTCCCGGGTCTTGTACACACCGCCCGTCAAGTCATGAAAGTCGGTAACACCTGAAGCCGGTGGCCCAACCCTTGTGGAGGGAGCCGTCGAAGGTGGGATCGGTAATTAGGACTAAGTCGT

*Gordonia amicalis:*

GGCTCAGGACGAACGCTGGCGGCGTGCTTAACACATGCAAGTCGAACGGAAAGGCCCGCTTGCGGGTACTCGAGTGGCGAACGGGTGAGTAACACGTGGGTGATCTGCCCTGGACTCTGGGATAAGCCTGGGAAACTGGGTCTAATACCGGATATGACCTTACATCGCATGGTGTTTGGTGGAAAGCTTTTGCGGTTCAGGATGGGCCCGCGGCCTATCAGCTTGTTGGTGGGGTAATGGCCTACCAAGGCGACGACGGGTAGCCGACCTGAGAGGGTGATCGGCCACACTGGGACTGAGACACGGCCCAGACTCCTACGGGAGGCAGCAGTGGGGAATATTGCACAATGGGCGCAAGCCTGATGCAGCGACGCCGCGTGAGGGATGACGGCCTTCGGGTTGTAAACCTCTTTCACCAGGGACGAAGCGCAAGTGACGGTACCTGGAGAAGAAGCACCGGCCAACTACGTGCCAGCAGCCGCGGTAATACGTAGGGTGCGAGCGTTGTCCGGAATTACTGGGCGTAAAGAGCTCGTAGGCGGTTTGTCGCGTCGTCTGTGAAATTCTGCAACTCAATTGTAGGCGTGCAGGCGATACGGGCAGACTTGAGTACTACAGGGGAGACTGGAATTCCTGGTGTAGCGGTGAAATGCGCAGATATCAGGAGGAACACCGGTGGCGAAGGCGGGTCTCTGGGTAGTAACTGACGCTGAGGAGCGAAAGCGTGGGTAGCGAACAGGATTAGATACCCTGGTAGTCCACGCCGTAAACGGTGGGTACTAGGTGTGGGGCTCATTTCACGAGTTCCGTGCCGTAGCTAACGCATTAAGTACCCCGCCTGGGGAGTACGGCCGCAAGGCTAAAACTCAAAGGAATTGACGGGGGCCCGCACAAGCGGCGGAGCATGTGGATTAATTCGATGCAACGCGAAGAACCTTACCTGGGTTTGACATACACCAGAAAGCTGTAGAGATATAGCCCCCCTTGTGGTTGGTGTACAGGTGGTGCATGGCTGTCGTCAGCTCGTGTCGTGAGATGTTGGGTTAAGTCCCGCAACGAGCGCAACCCTTGTCCTGTATTGCCAGCGGGTTATGCCGGGGACTTGCAGGAGACTGCCGGGGTCAACTCGGAGGAAGGTGGGGATGACGTCAAGTCATCATGCCCCTTATGTCCAGGGCTTCACACATGCTACAATGGCTGGTACAGAGGGCTGCGATACCGTGAGGTGGAGCGAATCCCTTAAAGCCAGTCTCAGTTCGGATTGGGGTCTGCAACTCGACCCCATGAAGTCGGAGTCGCTAGTAATCGCAGATCAGCAACGCTGCGGTGAATACGTTCCCGGGCCTTGTACACACCGCCCGTCACGTCATGAAAGTCGGTAACACCCGAAGCCGGTGGCCTAACCCTTGTGGAGGGAGCTGTCGAAGGTGGGATCGGCGATTGGGACGAAGTCGT
